# Supplementary material for: Asymmetric nuclear division in neural stem cells generates sibling nuclei that differ in size, envelope composition, and chromatin organization
Source: Curr Biol. 2021 Sep 27;31(18):3973–3983.e4. doi: 10.1016/j.cub.2021.06.063 (PMC8491657; doi:10.1016/j.cub.2021.06.063)
Supplement: Document S1. Figures S1–S6 [file mmc1.pdf]

Current Biology, Volume *31*

Supplemental Information

Asymmetric nuclear division in neural stem cells generates sibling nuclei that differ in size, envelope composition, and chromatin organization

Chantal Roubinet, Ian J. White, and Buzz Baum

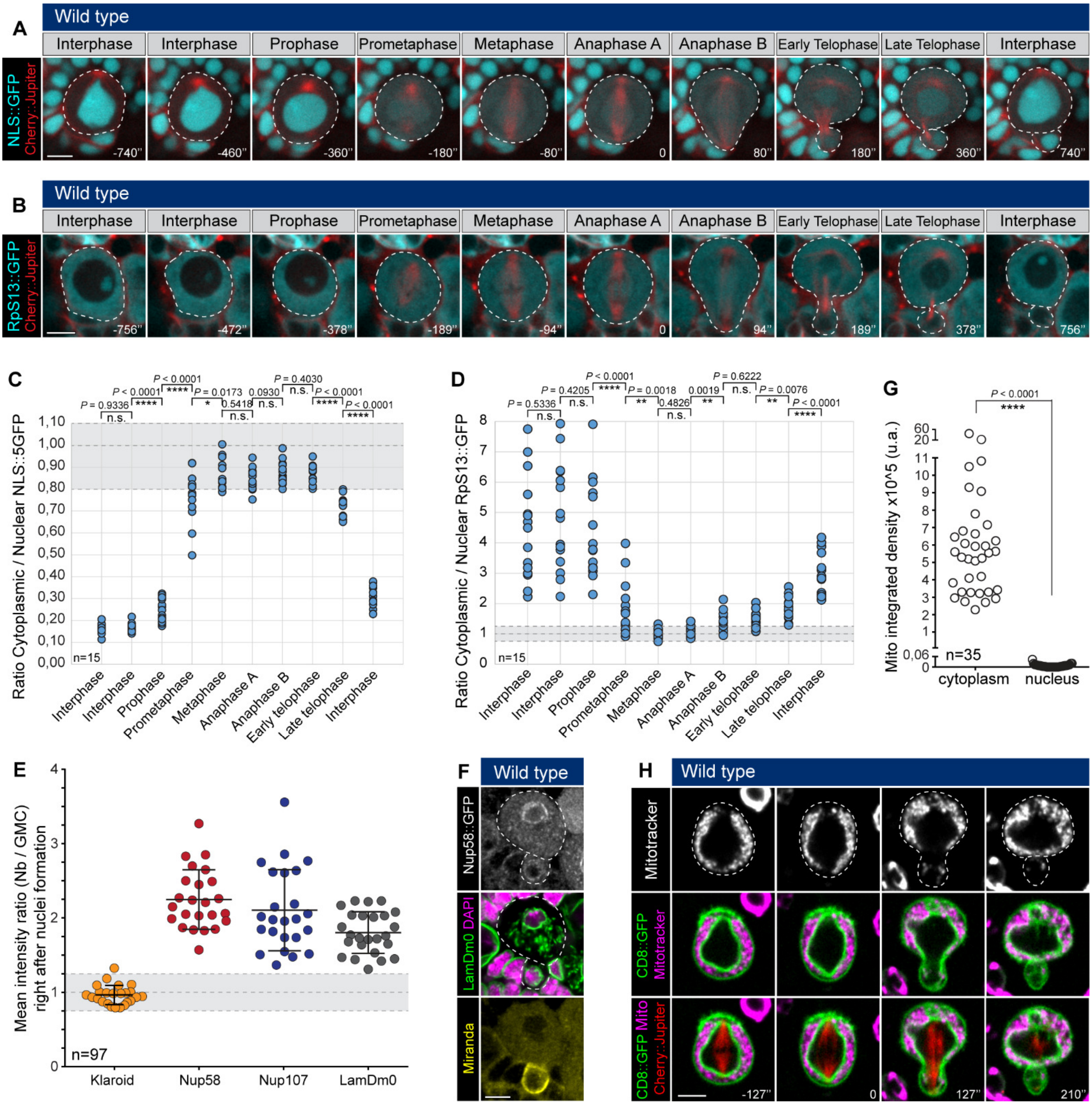

A
Wild type
Interphase
Interphase
Prophase
Prometaphase
Metaphase
Anaphase A
Anaphase B
Early Telophase
Late Telophase
Interphase
NLS::GFP
Cherry::Jupiter
-740''
-460''
-360''
-180''
-80''
0
80''
180''
360''
740''
B
Wild type
RpS13::GFP
Cherry::Jupiter
-756''
-472''
-378''
-189''
-94''
0
94''
189''
378''
756''
C
Ratio Cytoplasmic / Nuclear NLS::5GFP
n=15
D
Ratio Cytoplasmic / Nuclear RpS13::GFP
n=15
G
Mito integrated density x10^5 (u.a.)
n=35
cytoplasm
nucleus
P < 0.0001
E
Mean intensity ratio (Nb / GMC) right after nuclei formation
n=97
Klaroid
Nup58
Nup107
LamDm0
F
Wild type
Nup58::GFP
LamDm0 DAPI
Miranda
H
Wild type
Mitotracker
CD8::GFP Mitotracker
CD8::GFP Mito Cherry::Jupiter
-127''
0
127''
210''

Figure S1: The nuclear envelope of fly neuroblasts is maintained during mitosis and remodelled to generate two sibling nuclei that differ in size and composition. Related to Figure 1.

(**A-B**), Time lapse of dividing neuroblast expressing Cherry::Jupiter (red) and (**A**) NLS::5GFP (blue) or (**B**) RpS13::GFP (blue). (**C-D**) Mean intensity of (**C**) NLS::5GFP or (**D**) RpS13::GFP is measured in the cytoplasm and in the nuclear compartment for 10 time points throughout the cell cycle, then the ratio (cytoplasm/nuclear compartment) is plotted on the graph. The grey area corresponds to a ratio equal to 1 ± 0.25. Number of analysed cells = 15 for each condition. Asterisks denote statistical significance, derived from unpaired t tests: n.s.: not significant, *: $p \leq 0.05$, **: $p \leq 0.01$ and ****: $p \leq 0.0001$. (**E**) Graph showing the mean intensity ratio of Klaroid, Nup58, Nup107 and LamDm0 between the neuroblast and GMC nucleus. Bars indicate mean ± standard deviation. Number of analysed cells = 97. (**F**) Representative images of a neuroblast expressing Nup58::GFP (white on the top panel), fixed and stained for LamDm0 (green on the merge), DAPI (pink on the merge) and Miranda (yellow on the bottom panel). (**G**) Graph showing the integrated density of Mitotracker signal in the cytoplasm and in the nuclear compartment, during metaphase. Number of analysed cells = 35. Asterisks denote statistical significance, derived from unpaired t tests: ****: $p \leq 0.0001$. (**H**) Time lapse images of dividing neuroblast expressing Cherry::Jupiter (red on the merge), CD8::GFP (green on the merge) and incubated in presence of Mitotracker (white on the top panel, pink on the merges). For each experiment, the data were collected from at least 3 independent experiments. Scale bars are 5µm.

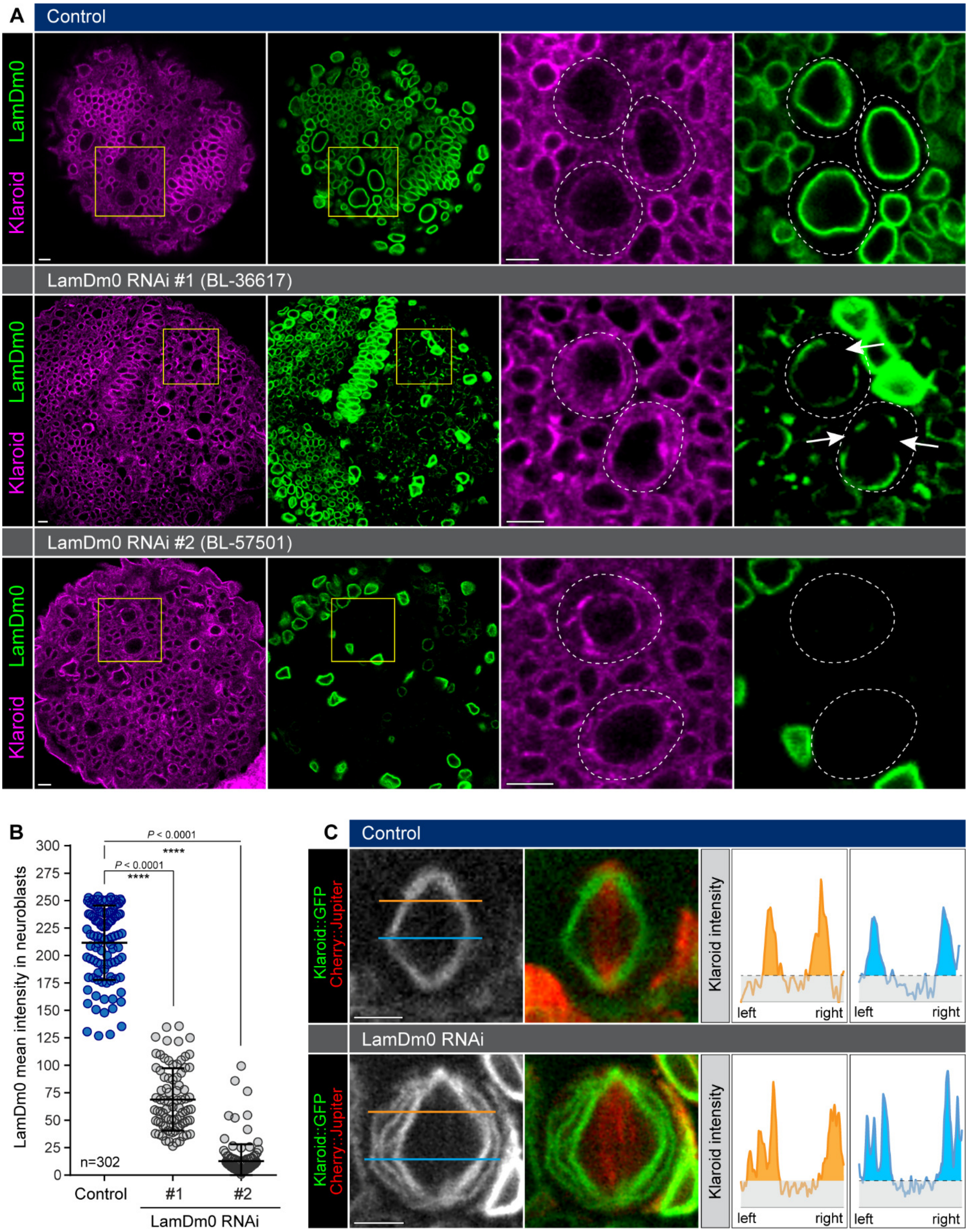

A
Control
LamDm0
Klaroid
LamDm0 RNAi #1 (BL-36617)
LamDm0 RNAi #2 (BL-57501)
B
P < 0.0001
****
LamDm0 mean intensity in neuroblasts
300
275
250
225
200
175
150
125
100
75
50
25
0
n=302
Control
#1
#2
LamDm0 RNAi
C
Klaroid::GFP
Cherry::Jupiter
Klaroid intensity
left
right
LamDm0 RNAi

Figure S2: A nuclear lamina is required for nuclear envelope maintenance during passage through mitosis. Related to Figure 2.

(**A**) Brain lobes expressing Klaroid (purple) in all cell types, Dicer in neuroblasts and their progeny (WorGal4 driver) (top panel) as well as an RNAi against LamDm0 in neuroblasts and their progeny (middle and bottom panels – RNAi #1 and #2, respectively), fixed and stained for LamDm0 (green). White dashed lines represent neuroblasts and white arrows show ruptures in the lamina. (**B**) Graph showing LamDm0 mean intensity along the nuclear envelope, in neuroblasts control and in neuroblasts expressing an RNAi against LamDm0 (RNAi #1 and #2). Bars indicate mean ± standard deviation. Number of analysed cells = 302. Asterisks denote statistical significance, derived from unpaired t tests: ****: $p \leq 0.0001$. (**C**) Representative images of a control neuroblast (top panel) and of a neuroblast expressing an RNAi against LamDm0 (bottom panel), expressing Klaroid::GFP (in white on the left, green on the merge) and Cherry::Jupiter (red on the merge). Graphs show plot profiles done along the orange and blue lines. For each experiment, the data were collected from at least 3 independent experiments.

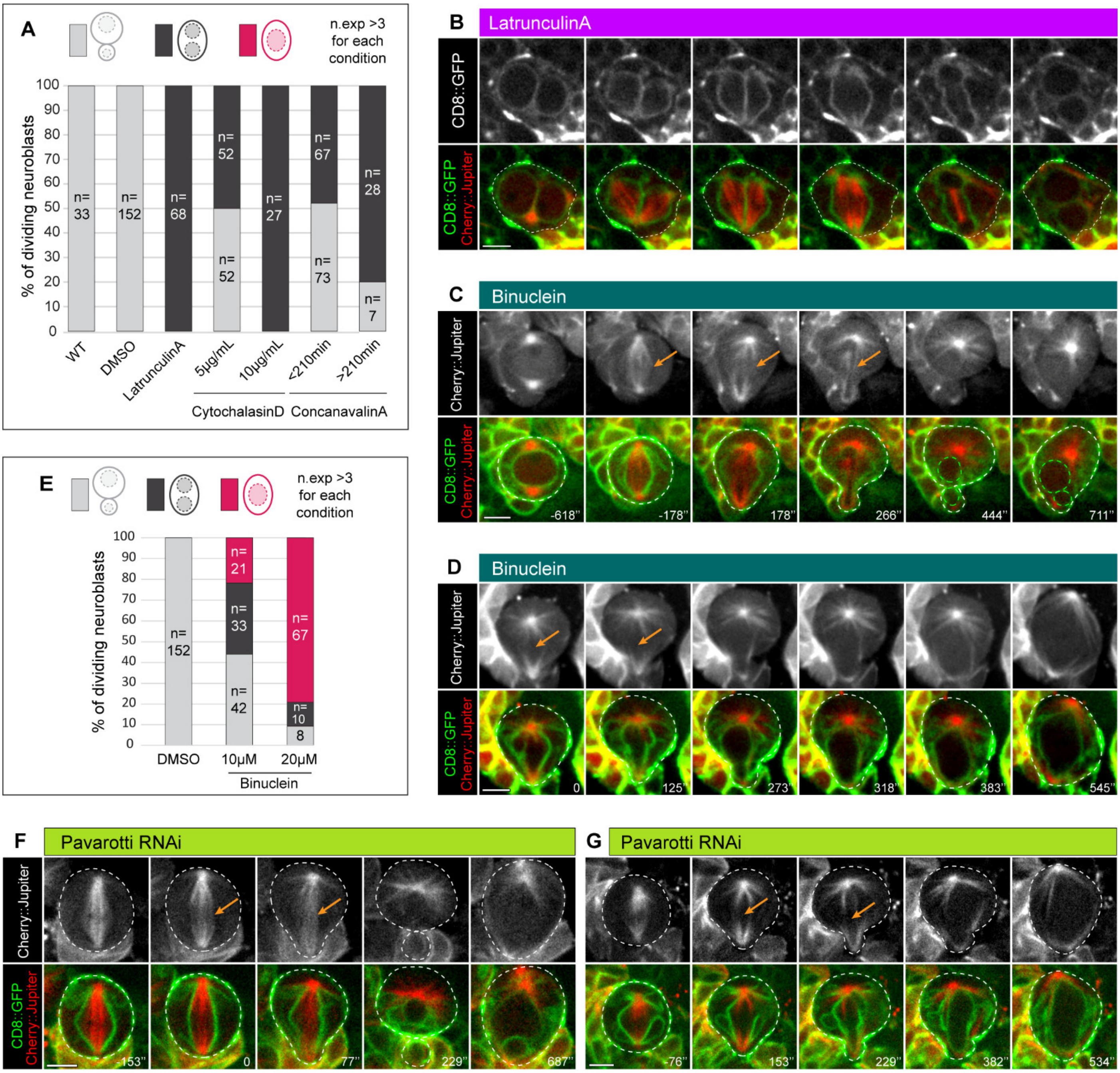

A
n.exp >3
for each
condition
% of dividing neuroblasts
100
90
80
70
60
50
40
30
20
10
0
n= 33
n= 152
n= 68
n= 52
n= 52
n= 27
n= 67
n= 73
n= 28
n= 7
WT
DMSO
LatrunculinA
5µg/mL
10µg/mL
<210min
>210min
CytochalasinD
ConcanavalinA
B
LatrunculinA
CD8::GFP
CD8::GFP Cherry::Jupiter
C
Binuclein
Cherry::Jupiter
CD8::GFP Cherry::Jupiter
-618''
-178''
178''
266''
444''
711''
D
Binuclein
Cherry::Jupiter
CD8::GFP Cherry::Jupiter
0
125''
273''
318''
383''
545''
E
n.exp >3
for each
condition
% of dividing neuroblasts
n= 152
n= 21
n= 33
n= 42
n= 67
n= 10
8
DMSO
10µM
20µM
Binuclein
F
Pavarotti RNAi
Cherry::Jupiter
CD8::GFP Cherry::Jupiter
-153''
0
77''
229''
687''
G
Pavarotti RNAi
-76''
153''
229''
382''
534''

Figure S3: Nuclear division is a sealing-dependent process. Related to Figure 3.

(**A**) Quantification of neuroblasts dividing normally (grey category), failing in cell division but still dividing their nucleus (black category) or failing in both cell and nuclear division (pink category). The number of analysed cells for each condition is indicated on the graph. (**B**) Representative timelapse images of binucleated neuroblast expressing CD8::GFP (white on the top panel, green on the merge) and Cherry::Jupiter (red on the merge), failing a second round of cell division. (**C**) Representative timelapse images of Binuclein-treated neuroblast expressing Cherry::Jupiter (white on the top panel, red on the merge) and CD8::GFP (green on the merge) failing only in cell division. Orange arrows show the central spindle. (**D**) Representative timelapse images of Binuclein-treated neuroblast expressing Cherry::Jupiter (white on the top panel, red on the merge) and CD8::GFP (green on the merge) failing in both cell and nuclear division. Orange arrows show the absence of a central spindle. (**E**) Quantification of neuroblasts dividing normally (grey category), failing in cell division but still undergoing nuclear division (black category) or failing in both cell and nuclear division (pink category) after DMSO or Binuclein treatment. The number of cells analysed for each condition is indicated on the graph. (**F, G**) Representative time lapse images of neuroblasts expressing an RNAi against Pavarotti, CD8::GFP (green on the merge) and Cherry::Jupiter (white on the top panel, red on the merge), failing only in cell division (**F**) or in both cell and nuclear division (**G**). Orange arrows show the presence (**F**) or absence (**G**) of central spindle. For each experiment, the data were collected from at least 3 independent experiments. Scale bar is 5µm.

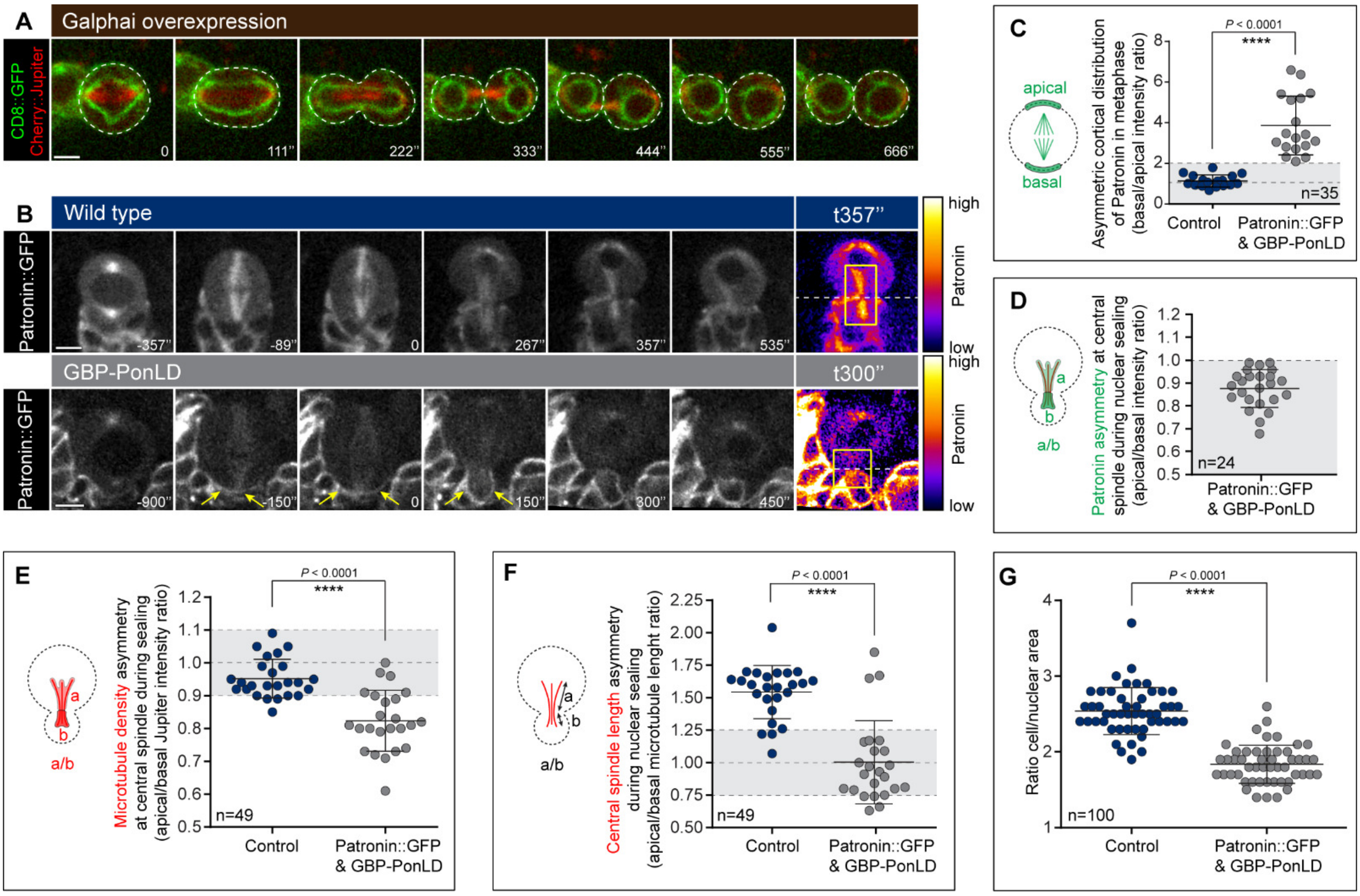

A
Galphai overexpression
CD8::GFP Cherry::Jupiter
0
111''
222''
333''
444''
555''
666''
B
Wild type
t357''
high
Patronin
low
Patronin::GFP
-357''
-89''
0
267''
357''
535''
GBP-PonLD
t300''
-900''
-150''
0
150''
300''
450''
C
apical
basal
Asymmetric cortical distribution of Patronin in metaphase (basal/apical intensity ratio)
P < 0.0001
****
n=35
Control
Patronin::GFP & GBP-PonLD
D
a
b
a/b
Patronin asymmetry at central spindle during nuclear sealing (apical/basal intensity ratio)
n=24
Patronin::GFP & GBP-PonLD
E
Microtubule density asymmetry at central spindle during sealing (apical/basal Jupiter intensity ratio)
P < 0.0001
****
n=49
Control
Patronin::GFP & GBP-PonLD
F
Central spindle length asymmetry during nuclear sealing (apical/basal microtubule lenght ratio)
P < 0.0001
****
n=49
Control
Patronin::GFP & GBP-PonLD
G
Ratio cell/nuclear area
P < 0.0001
****
n=100
Control
Patronin::GFP & GBP-PonLD

Figure S4: Asymmetric nuclear division depends on nuclear sealing at sites dictated by the spindle. Related to Figure 4.

(**A**) Representative time lapse images of a neuroblast overexpressing Galphai, CD8::GFP (green) and Cherry::Jupiter (red). (**B**) Representative time lapse images of a neuroblast expressing Patronin::GFP alone (top panel) or Patronin::GFP together with the localization domain of Pon fused to GFP Binding Protein (GBP-PonLD) (bottom panel). Yellow arrows show the basal recruitment of Patronin::GFP. The last pictures correspond to the sealing time (time point 357" and 300", respectively), and is shown with a gradient of colour to highlight the depletion of Patronin on the apical half of the central spindle after co-expression with GBP-PonLD. (**C**) Graph showing Patronin distribution at the apical versus basal cortex, in metaphase, in control neuroblasts and in neuroblasts expressing Patronin::GFP together with GBP-PonLD. Bars indicate mean ± standard deviation. Number of analysed cells = 35. Asterisks denote statistical significance, derived from unpaired t tests: ****: $p \leq 0.0001$. (**D**) Graph showing Patronin distribution at the apical versus basal half of the central spindle. Bars indicate mean ± standard deviation. Number of analysed cells = 24. (**E**) Graph showing the microtubule density ratio of the central spindle (apical versus basal half) in control neuroblasts and in neuroblasts expressing Patronin::GFP and GBP-PonLD. Number of analysed cells = 49. Bars indicate mean ± standard deviation. Asterisks denote statistical significance, derived from unpaired t tests: ****: $p \leq 0.0001$. (**F**) Graph showing the microtubule length ratio of the central spindle (apical versus basal half) in control neuroblasts and in neuroblasts expressing Patronin::GFP and GBP-PonLD. Number of analysed cells = 49. Bars indicate mean ± standard deviation. Asterisks denote statistical significance, derived from unpaired t tests: ****: $p \leq 0.0001$. (**G**) Graph showing the ratio between cell and nuclear size in control neuroblasts and in neuroblasts expressing Patronin::GFP and GBP-PonLD. Number of analysed cells = 100. Bars indicate mean ± standard deviation. Asterisks denote statistical significance, derived from unpaired t tests: ****: $p \leq 0.0001$. For each experiment, the data were collected from at least 3 independent experiments. Scale bars are 5µm.

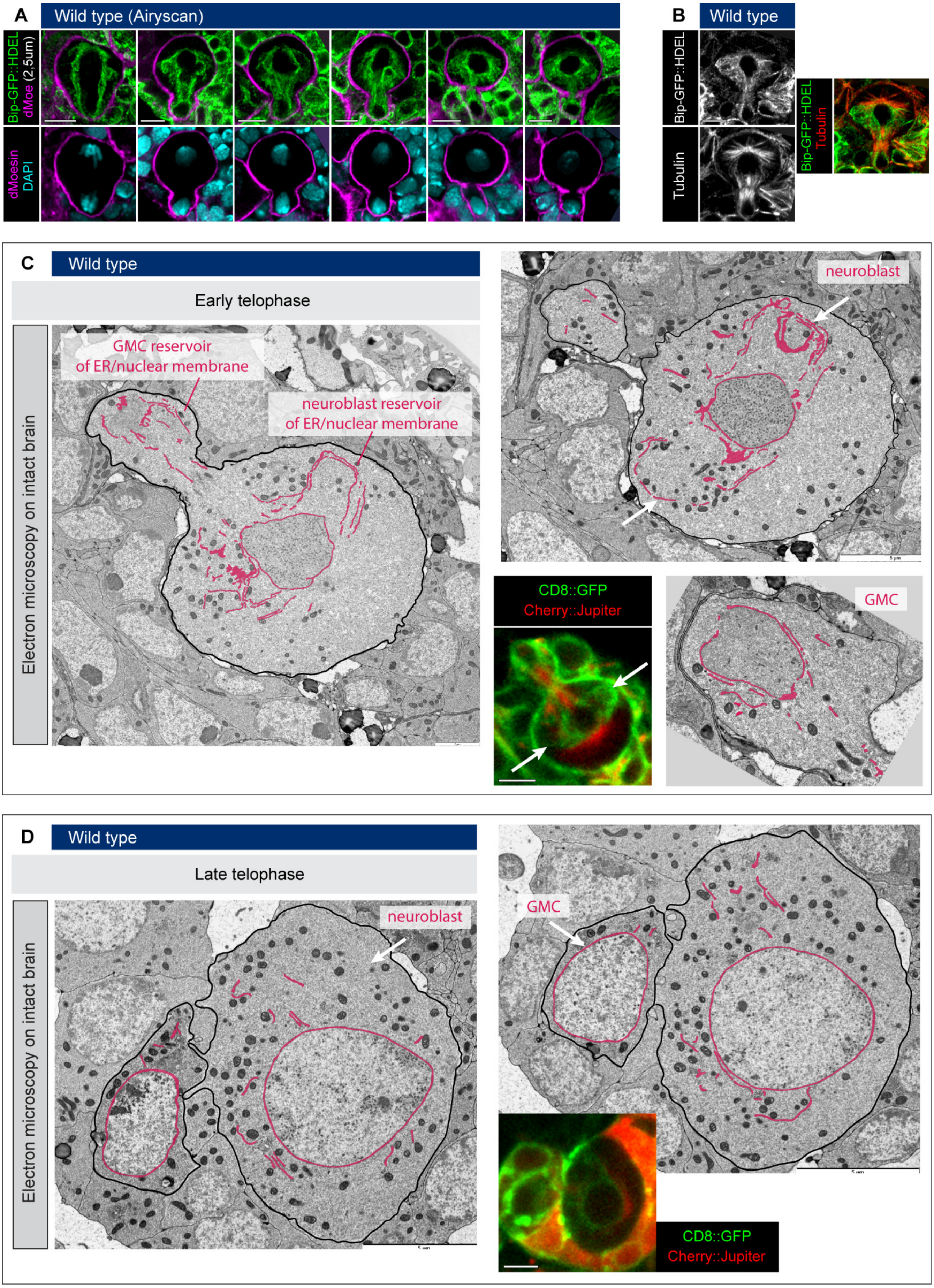
A
Wild type (Airyscan)
Bip-GFP::HDEL dMoe (2,5um)
dMoesin DAPI
B
Wild type
Bip-GFP::HDEL
Tubulin
Bip-GFP::HDEL Tubulin
C
Wild type
Early telophase
Electron microscopy on intact brain
GMC reservoir of ER/nuclear membrane
neuroblast reservoir of ER/nuclear membrane
neuroblast
CD8::GFP
Cherry::Jupiter
GMC
D
Wild type
Late telophase
Electron microscopy on intact brain
neuroblast
GMC
CD8::GFP
Cherry::Jupiter

Figure S5: Final nuclear size are achieved via differential growth of the two daughter nuclei. Related to Figure 5.

(**A**) Representative Airyscan images of neuroblasts expressing the ER marker Bip-SfGFP::HDEL (green) stained for the cortical marker dMoesin (pink) and DAPI (blue). Maximal projection of 2.5 um. (**B**) Representative Airyscan images of neuroblast expressing the ER marker Bip-SfGFP::HDEL (white on the top panel, green on the merge) stained for Tubulin (white on the bottom panel, red on the merge. (**C**) Correlative Light and Electron Microscopy showing the asymmetric partitioning of ER/nuclear membrane reservoir between the two daughter cells, during early telophase. (**D**) Correlative Light and Electron Microscopy performed in late telophase, after nuclear growth. Note the very small remaining of nuclear membranes reservoir into the neuroblast, compared with mitotic neuroblasts in late anaphase/early telophase (Figure 5F, Figure S5C).

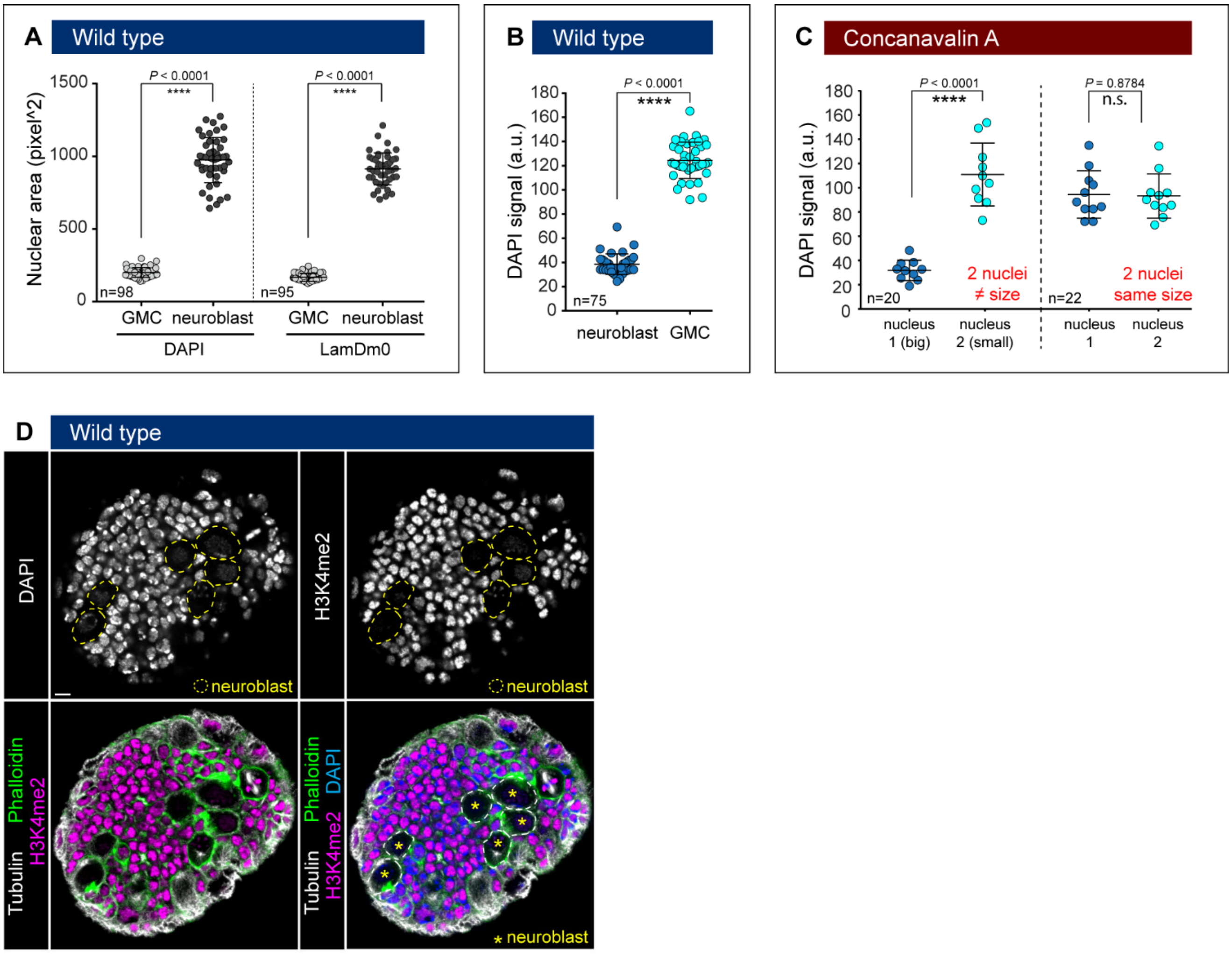

Figure S6: Asymmetric nuclear division affects chromatin organization. Related to Figure 6.

(**A**) Graph showing nuclear area of GMC and neuroblast nucleus based on DAPI (left) and LamDm0 (right) staining. Number of analysed cells = 98 and 95 for DAPI and LamDm0 staining, respectively. Bars indicate mean ± standard deviation. Asterisks denote statistical significance, derived from unpaired t tests: ****: p ≤ 0.0001. (**B**) Graph showing the DAPI mean intensity signal in the neuroblast and GMC nucleus. Number of analysed cells = 75. Bars indicate mean ± standard deviation. Asterisks denote statistical significance, derived from unpaired t tests: ****: p ≤ 0.0001. (**C**) Graph showing the DAPI mean intensity signal in sibling nuclei of binucleated neuroblasts. Number of analysed cells = 42. Bars indicate mean ± standard deviation. Asterisks denote statistical significance, derived from unpaired t tests: n.s.: not significant, ****: p ≤ 0.0001. (**D**) Wild type brain lobe fixed and stained for DAPI (white on the top left and blue on the merge), H3K4me2 (white on the top right and pink on the merges), Tubulin (white on the merges) and Phalloidin (green on the merges). Yellow dashed lines and yellow stars represent neuroblasts. For each experiment, the data were collected from at least 3 independent experiments.
