# Supplementary material for: Asymmetric nuclear division in neural stem cells generates sibling nuclei that differ in size, envelope composition, and chromatin organization
Source: Curr Biol. 2021 Sep 27;31(18):3973–3983.e4. doi: 10.1016/j.cub.2021.06.063 (PMC8491657; doi:10.1016/j.cub.2021.06.063)
Supplement: Document S2. Article plus supplemental information [file mmc9.pdf]

Current Biology

Article

Asymmetric nuclear division in neural stem cells generates sibling nuclei that differ in size, envelope composition, and chromatin organization

Graphical abstract

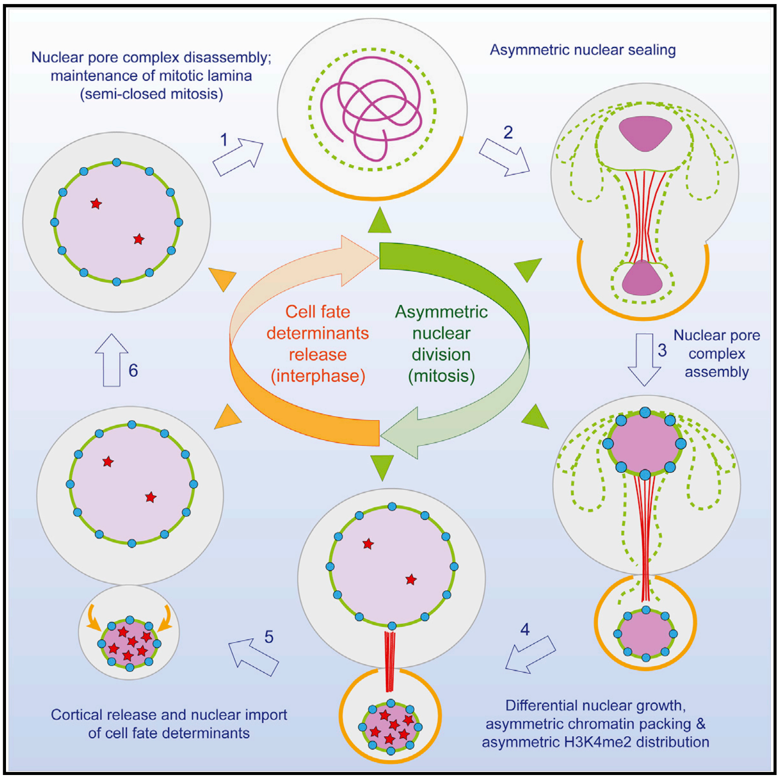

Highlights

- Neural stem cells maintain a nuclear lamina and envelope throughout mitosis
- Asymmetric nuclear division forms sibling nuclei that differ in size and composition
- The nuclear asymmetry depends on an asymmetric spindle and differential growth
- Asymmetric nuclear division likely contributes to the generation of cell diversity

Authors

Chantal Roubinet, Ian J. White, Buzz Baum

Correspondence

c.roubinet@ucl.ac.uk (C.R.), bbaum@mrc-lmb.cam.ac.uk (B.B.)

In brief

Roubinet et al. show that fly neural stem cells maintain a mitotic nuclear compartment throughout mitosis. This mitotic nuclear envelope is remodeled in a two-step process to give rise to sibling nuclei that differ in size and envelope composition prior to the nuclear import of cell fate determinants, potentially biasing daughter cell fates.

Roubinet et al., 2021, Current Biology *31*, 3973–3983
September 27, 2021 © 2021 The Author(s). Published by Elsevier Inc.
https://doi.org/10.1016/j.cub.2021.06.063

CellPress

Current Biology

CellPress
OPEN ACCESS

Article

Asymmetric nuclear division in neural stem cells generates sibling nuclei that differ in size, envelope composition, and chromatin organization

Chantal Roubinet,[1,3,4,*] Ian J. White,[1] and Buzz Baum[1,2,3,*]
[1]MRC Laboratory for Molecular Biology, University College London, London, UK
[2]Institute for the Physics of Living Systems, University College London, London, UK
[3]MRC Laboratory of Molecular Cell Biology, Cambridge, UK
[4]Lead contact
*Correspondence: c.roubinet@ucl.ac.uk (C.R.), bbaum@mrc-lmb.cam.ac.uk (B.B.)
https://doi.org/10.1016/j.cub.2021.06.063

SUMMARY

Although nuclei are the defining features of eukaryotes, we still do not fully understand how the nuclear compartment is duplicated and partitioned during division. This is especially the case for organisms that do not completely disassemble their nuclear envelope upon entry into mitosis. In studying this process in *Drosophila* neural stem cells, which undergo asymmetric divisions, we find that the nuclear compartment boundary persists during mitosis thanks to the maintenance of a supporting nuclear lamina. This mitotic nuclear envelope is then asymmetrically remodeled and partitioned to give rise to two daughter nuclei that differ in envelope composition and exhibit a >30-fold difference in volume. The striking difference in nuclear size was found to depend on two consecutive processes: asymmetric nuclear envelope resealing at mitotic exit at sites defined by the central spindle, and differential nuclear growth that appears to depend on the available local reservoir of ER/nuclear membranes, which is asymmetrically partitioned between the two daughter cells. Importantly, these asymmetries in size and composition of the daughter nuclei, and the associated asymmetries in chromatin organization, all become apparent long before the cortical release and the nuclear import of cell fates determinants. Thus, asymmetric nuclear remodeling during stem cell divisions may contribute to the generation of cellular diversity by initiating distinct transcriptional programs in sibling nuclei that contribute to later changes in daughter cell identity and fate.

INTRODUCTION

Nuclei are the defining feature of the eukaryotic cell. Although nuclear division—the process by which a nucleus divides into two—was one of the first subjects studied by Walther Flemming and other pioneers of cell biology, it remains one of the most poorly understood aspects of the division process. While many eukaryotic cells exhibit a so-called "open mitosis," in which the nuclear envelope is disassembled at mitotic entry before being reformed anew around chromosomes at mitotic exit,[1–5] other eukaryotes undergo a "closed mitosis" in which they maintain an intact nuclear compartment and a diffusion barrier throughout.[3,6] At first sight, the mechanisms of open and closed nuclear division appear completely different. However, we recently identified a role for nuclear pore disassembly in the final steps of bridge scission during nuclear division in fission yeast—a classic model of closed mitosis.[7,8] This finding suggests that the mechanisms of open and closed mitosis differ, at least in part, as a simple consequence of shifts in the timing and position of nuclear pore disassembly. In line with open and closed mitosis sitting at either ends of a spectrum, the mechanism of nuclear division in many organisms such as flies and worms appears midway between these two extremes and is often described as a "semi-open" or "semi-closed" mitosis.[9–11] In addition, the way nuclei divide depends on cell type. Thus, the nuclear envelope is disassembled during mitosis in *Drosophila* cells in culture,[12] but is maintained in the syncytial fly embryo.[9,13,14] To further investigate the mechanism of nuclear division in an intermediate "semi-closed mitosis," we sought to study the process in *Drosophila* neural stem cells (neuroblasts), which divide asymmetrically to generate a large self-renewing neuroblast containing a large nucleus and a smaller differentiating ganglion mother cell (GMC) with a small nucleus.[15–17] However, it is not known how the nuclear compartment is partitioned during these stem cell divisions, nor how nuclear division might contribute to the formation of sibling nuclei with different transcriptional profiles/identities.[18,19] In this paper we show that the nuclear envelope of neuroblasts undergoes an asymmetric remodeling during mitosis, so that the genome is partitioned into daughter nuclei that differ by more than 30-fold in volume and in envelope composition. This analysis also reveals the emergence of differences in chromatin organization between the two daughter nuclei that precede the cortical release and nuclear

This is an open access article under the CC BY license (http://creativecommons.org/licenses/by/4.0/).

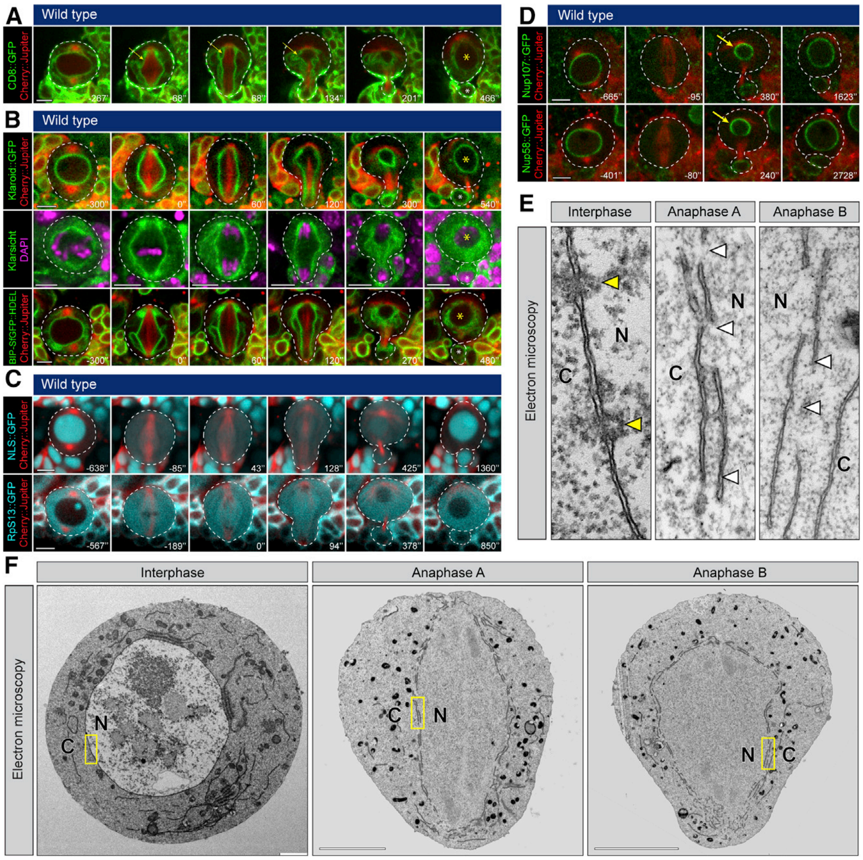

Figure 1. The nuclear envelope of fly neuroblasts is maintained during mitosis and remodeled to generate two sibling nuclei that differ in size and envelope composition

(A–D) Mitotic neuroblasts expressing Cherry::Jupiter in combination with either (A) CD8::GFP, (B) Klaroid::GFP, Klarsicht::GFP or BIP-SfGFP::HDEL, (C) NLS::5GFP or Rps13::GFP, or (D) Nup107::GFP or Nup58::GFP. Yellow arrows indicate higher nucleoporin density.

(E and F) Electron microscopy performed at various cell cycle stages: (E) magnified regions are marked in yellow in (F); yellow arrowheads show nuclear pores; white arrowheads show holes in the nuclear envelope; N, nuclear compartment; C, cytoplasm. Scale bars are 5 μm.

See also Figure S1 and Videos S1, S2, S3, and S4.

import of cell fate determinants. This suggests an important role for nuclear division in the generation of cellular diversity.

RESULTS

The nuclear envelope of fly neuroblasts is maintained during mitosis and remodeled to generate two sibling nuclei that differ in size and envelope composition

To characterize nuclear division in *Drosophila* neuroblasts, we used markers to label plasma and nuclear membranes (CD8),[20] the INM (Klaroid), ONM (Klarsicht) and the ER (Bip-SfGFP::HDEL). This identified a continuous membrane boundary enveloping the spindle throughout mitosis (Figures 1A and 1B; Videos S1 and S2), which underwent asymmetric remodeling at mitotic exit to give rise to two nuclei that differ in size (yellow versus white stars). Despite the presence of this bounding membrane, the nuclear-cytoplasmic diffusion barrier was lost at prometaphase before being re-established in the two daughter nuclei in late telophase, as measured by the distribution of nuclear (NLS::GFP) and cytoplasmic (RpS13::GFP) markers (Figures 1C and S1A–S1D and Videos S3 and S4). In line with this, nucleoporins (Nup58::GFP and Nup107::GFP) were lost from the nuclear membrane upon entry into mitosis, then recruited back in an asymmetric fashion to the newly formed nuclei at mitotic exit (Figure 1D, yellow arrows; Figure S1E) prior to the cortical release of polarity proteins such as Miranda (Figure S1F). These changes to the nuclear envelope were confirmed by electron microscopy. Thus, this ultrastructural analysis revealed the formation of highly fenestrated and multilayered mitotic nuclear envelope that lacks NPCs (Figures 1E and 1F). Nevertheless, while ribosomes were seen in both compartments during mitosis (Figure 1C, bottom panel, and 1E), larger structures such as vesicles and mitochondria were effectively excluded from the mitotic nuclear compartment (Figures 1F, S1G, and S1H). These data show that *Drosophila* neuroblasts undergo a semi-closed mitosis, characterized by the complete disassembly of NPCs and the reorganization of the nuclear envelope into multilayered fenestrated sheets.

A nuclear lamina is required for nuclear envelope maintenance during passage through mitosis

In the search for structural elements that might distinguish this type of division from a classic open mitosis,[21] we imaged cells expressing GFP-LamDm0, the sole B-type Lamin in the *Drosophila* genome. This revealed that, while the levels of LamDm0 present at the nuclear envelope were visibly reduced by late mitosis (1.8-fold), a lamina was maintained throughout (Figures 2A–2C and Video S5)—an observation confirmed using antibodies raised against endogenous LamDm0 (Figure 2D) or a form of endogenous LamDm0 that lacks phosphorylation on Serine 25 (Figure 2E).

When LamDm0 was silenced in neuroblasts and their progeny via RNAi, we found that mild reductions in LamDm0 levels were accompanied by discontinuities in the mitotic nuclear lamina (Figure S2A, middle panel, and S2B); whereas more complete silencing (Figure S2A, bottom panel, and S2B) led to the separation of nuclear membrane layers that fan out into the cytoplasm (Figures 2F and S2C). Similar nuclear membrane spreading was observed using correlative light and electron microscopy (CLEM) (Figures 2G and 2H, yellow arrows), which was associated with large discontinuities in the nuclear boundary (green arrows). Furthermore, more than 80% of mitotic neuroblasts depleted in LamDm0 underwent an abnormal nuclear division (Figure 2I), which included a dramatic delay in the onset of anaphase, the

CellPress
OPEN ACCESS

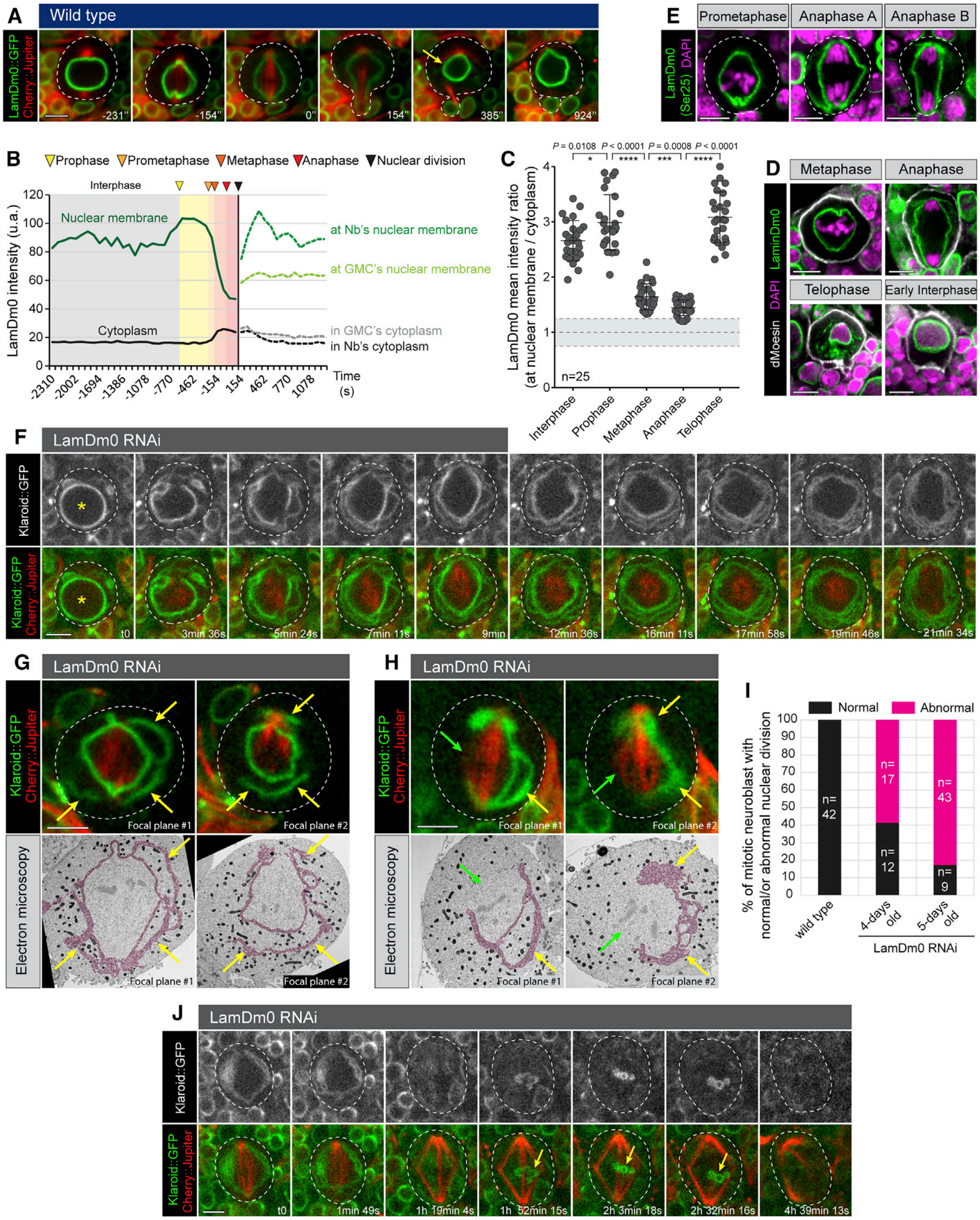

Figure 2. A nuclear lamina is required for nuclear envelope maintenance during passage through mitosis

(A) Mitotic neuroblast expressing LamDm0::GFP with Cherry::Jupiter. Yellow arrows point the higher LamDm0 density.

(B) LamDm0::GFP mean intensity along the nuclear membrane and in the cytoplasm, throughout the cell cycle.

(C) LamDm0 mean intensity ratio between the nuclear membrane and the cytoplasm is measured at 5 different stages for 25 cells. Grey area corresponds to a ratio of 1 ± 0.25. Bars indicate mean ± standard deviation. Asterisks denote statistical significance, derived from unpaired t tests: *$p \leq 0.05$, ***$p \leq 0.001$, and ****$p \leq 0.0001$.

(D) Immunostaining for LamDm0, dMoesin, and DAPI.

(E) Immunostaining for non-phosphorylated LamDm0 on serine 25 and DAPI.

(F) Representative time lapse of neuroblast expressing Klaroid::GFP, Cherry::Jupiter, and an RNAi against LamDm0. Yellow stars indicate presence of intact prophase nuclear compartment.

(legend continued on next page)

formation of defective spindles, a failure in chromosome segregation and cell division (Figure 2J), and an inability to reform nuclei at mitotic exit once nuclear membrane layers dispersed into the cytoplasm (Figure 2J). Together, these data show that LamDm0 depletion is sufficient to convert a semi-closed mitosis into an open one.

Asymmetric nuclear division depends on asymmetric nuclear sealing at sites dictated by the spindle

While live cell imaging of the nuclear envelope and plasma membrane during mitosis suggested that their remodeling might be causally linked—since both compartments developed a similar apico-basal asymmetry (Figures 3A and 3B), elongated at a similar rate (Figure 3C), and narrowed at the same position prior to cytokinesis (Figure 3D)—we were able to uncouple cell and nuclear division by perturbing cytokinesis using the actin-poisons Latrunculin A and Cytochalasin D, or by culturing isolated neuroblasts on Concanavalin A-coated dishes which interferes with furrow contraction.[22] Although many cells under these conditions ultimately failed in cytokinesis, all were able to divide their nucleus into two (n = 242; Figures 3E and S3A and Video S6), even after successive cytokinesis failures (Figure S3B). These observations rule out a model in which nuclear division is driven by local contraction of the cleavage furrow. Instead, high temporal resolution movies revealed that nuclear membranes elongated at both extremities of the central spindle, to seal the nuclear compartment to form two daughter nuclei (Figures 3F and 4A and Video S6). To test whether the central spindle is required for nuclear sealing, as suggested by this analysis, we used Binuclein 2 and RNAi to interfere with the function of the Aurora B kinase, a key regulator of the central spindle assembly.[23] In these experiments, two daughter nuclei were formed when a central spindle was visible (Figure S3C), leading to the generation of binucleated cells after cleavage furrow regression, while the lack of a central spindle was associated with a failure of both cell and nuclear division (Figures S3D, S3E, 3G, and 3H) . Similarly, following partial depletion of Pavarotti or Tumbleweed, two core components of the centralspindlin complex, nuclear division proceeded in cells with a central spindle (Figures 3G, 3I, and S3F), but failed when central spindle formation was compromised (Figures 3G, 3J, and S3G).

If nuclei are generated via a process of "sealing," how is the asymmetry in the size of the nascent nuclei generated? A first clue came from a quantitative analysis of live imaging data, which revealed a strong correlation between the asymmetry in the spindle and the sites of nuclear sealing (Figures 4B–4D; Pearson coefficient of 0.9878). However, to rule out a role for the asymmetry in daughter cell size in this process, we needed to uncouple the spindle asymmetry from the cell division asymmetry.[24] To do so, we overexpressed the polarity protein Gαi. While most of these cells divided symmetrically (Figure S4A), a subset of Gαi-expressing cells divided asymmetrically despite having near symmetric spindles (Figure 4E; cell division asymmetry ratio of 1.67 and spindle asymmetry ratio of 1.18). Strikingly, nuclear division in these cells was close to symmetric (Figure 4E; nuclear asymmetry ratio of 1.16). Thus, the size of daughter nuclei immediately after nuclear sealing appeared defined by the asymmetry of the spindle (Figure 4F, left graph; r = 0.9893), rather than by the asymmetry of cell division (Figure 4F, right graph; r = 0.6384). For the converse experiment, we selectively perturbed the normal asymmetry in central spindle length by co-expressing the microtubule-binding protein Patronin::GFP together with a nanobody fused to the basal localization domain of Pon (GBP-PonLD) and Cherry::Jupiter. While Patronin::GFP was uniformly distributed along the central spindle when expressed alone (Figure S4B, top, t357″ insert), the co-expression of GBP-PonLD was sufficient to redistribute Patronin::GFP to the basal cortex (Figure S4C) and to the basal part of the central spindle (Figure S4B, bottom, t300″ insert and Figure S4D). This led to a local increase in microtubule density along the basal part of the central spindle (apical/basal Jupiter intensity ratio: 0.82 ± 0.09 versus 0.95 ± 0.06 for control cells) (Figure S4E) and induced a 1.5-fold change in the length of the central spindle relative to that found in the control (apical/basal length ratio: 1.54 ± 0.21 versus 1 ± 0.32 for control cells) (Figure S4F). This led to an increase of spindle asymmetry (Figure 4I, left graph) and a bias in the sites of nuclear sealing (Figure 4G versus 4H, green dashed lines; Figure 4I, right graph). As a result, the asymmetry of the nuclear division was increased without changing the position of the cytokinetic furrow, altering the cell/nuclear size ratio (Figure S4G). Together, these data show that it is the asymmetry in the spindle that biases re-sealing of the nuclear compartment to generate nascent sibling nuclei with different size.

Final nuclear sizes are achieved via differential growth of the two daughter nuclei

This initial bias in nuclear size was dramatically increased during telophase/early G1. Thus, neuroblast nuclei had a volume ~4.5 times higher than GMC nuclei at the time of nuclear sealing but were ~37-fold larger by the time nuclear size stabilized (Figure 5A). In fact, while the size of GMC nuclei remained approximately unchanged following nuclear sealing (Figure 5B, steps I and II), neuroblast nuclei grew extensively (Figure 5B, step II) until they reached a stable size (Figure 5B, step III). Interestingly, sealing events occurring on the lateral sides of nuclei (Figure 4A, orange arrows), appeared to fold in a cytoplasmic reservoir of membrane labeled with both INM and ER markers (Klaroid::GFP and Bip-SfGFP::HDEL) (Figures 4A, 5C, 5D, S5A, and S5B). These membranes were 20 times more abundant in neuroblasts relative to the GMC right at the time of nuclear sealing (Figure 5E). Furthermore, this pool of membrane was progressively depleted as neuroblast nuclei grew and was near absent by the time nuclear size was stabilized in late telophase, as visualized by both light microscopy and CLEM (Figures 5F and S5C versus

(G and H) CLEM showing progressive dispersion of nuclear membranes into the cytoplasm (G) and a loss of nuclear compartment integrity (H) after LamDm0 RNAi expression.
(I) Quantification of mitotic neuroblasts expressing an RNAi against LamDm0 and displaying normal or abnormal nuclear division. Number of analyzed cells = 123.
(J) Representative time-lapse images of LamDm0 RNAi neuroblast expressing Klaroid::GFP and Cherry::Jupiter, in which nuclear membranes disperse and nuclei fail to reform at mitotic exit. At least 3 independent experiments were performed.
Scale bars are 5 μm. See also Figure S2 and Video S5.

CellPress
OPEN ACCESS

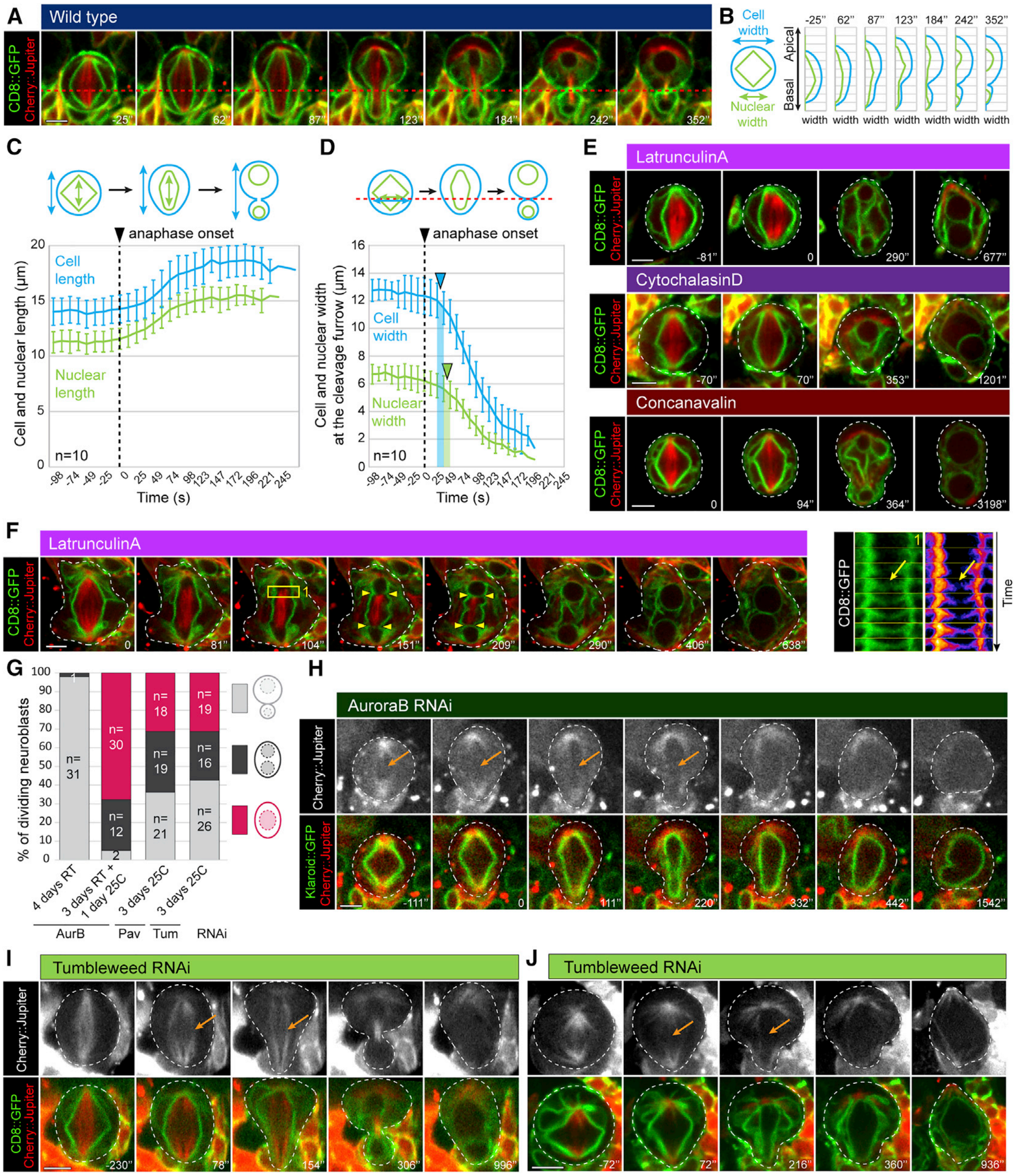

Figure 3. Nuclear division is a sealing-dependent process

(A) Dividing neuroblasts expressing Cherry::Jupiter and CD8::GFP. The red dashed line represents the site of cortical furrowing.

(B) Graph showing the cell and nuclear width from the apical to the basal cortex, for 7 time points.

(C) Graph showing the cell and nuclear length along the apico-basal axis for ten cells throughout mitosis. Error bars represent standard deviation and the dashed line indicates anaphase onset.

(D) Graph showing the cell and nuclear width at the cleavage furrow region throughout mitosis, averaged from ten cells. Error bars represent standard deviation, dashed line indicates anaphase onset, and blue and green arrowheads indicate start of cortical and nuclear furrowing, respectively.

(E) Representative time lapse sequences of mitotic neuroblasts expressing CD8::GFP and Cherry::Jupiter, and failing in cytokinesis after LatrunculinA, CytochalasinD, or ConcanavalinA treatment.

(F) Time-lapse images of a Latrunculin-treated neuroblast expressing CD8::GFP and Cherry::Jupiter. The yellow square indicates area used on the kymograph and the yellow arrowheads represent sites of nuclear sealing.

(G) Quantification of dividing neuroblasts, neuroblasts failing in cell division but not in nuclear division, or failing in both cell and nuclear division after expression of an RNAi against AuroraB, Pavarotti, or Tumbleweed. The number of cells analyzed for each condition is indicated.

(H) Representative time-lapse images of neuroblast expressing an RNAi against AuroraB, CD8::GFP, and Cherry::Jupiter, failing in both cell and nuclear division. Orange arrow shows the absence of central spindle.

(I and J) Representative time-lapse images of neuroblasts expressing an RNAi against Tumbleweed, CD8::GFP, and Cherry::Jupiter, failing cell division (I), or failing in both cell and nuclear division (J). Orange arrows show the presence (I) or absence (J) of central spindle. For each experiment, n ≥ 3.

Scale bars are 5 μm. See also Figure S3 and Video S6.

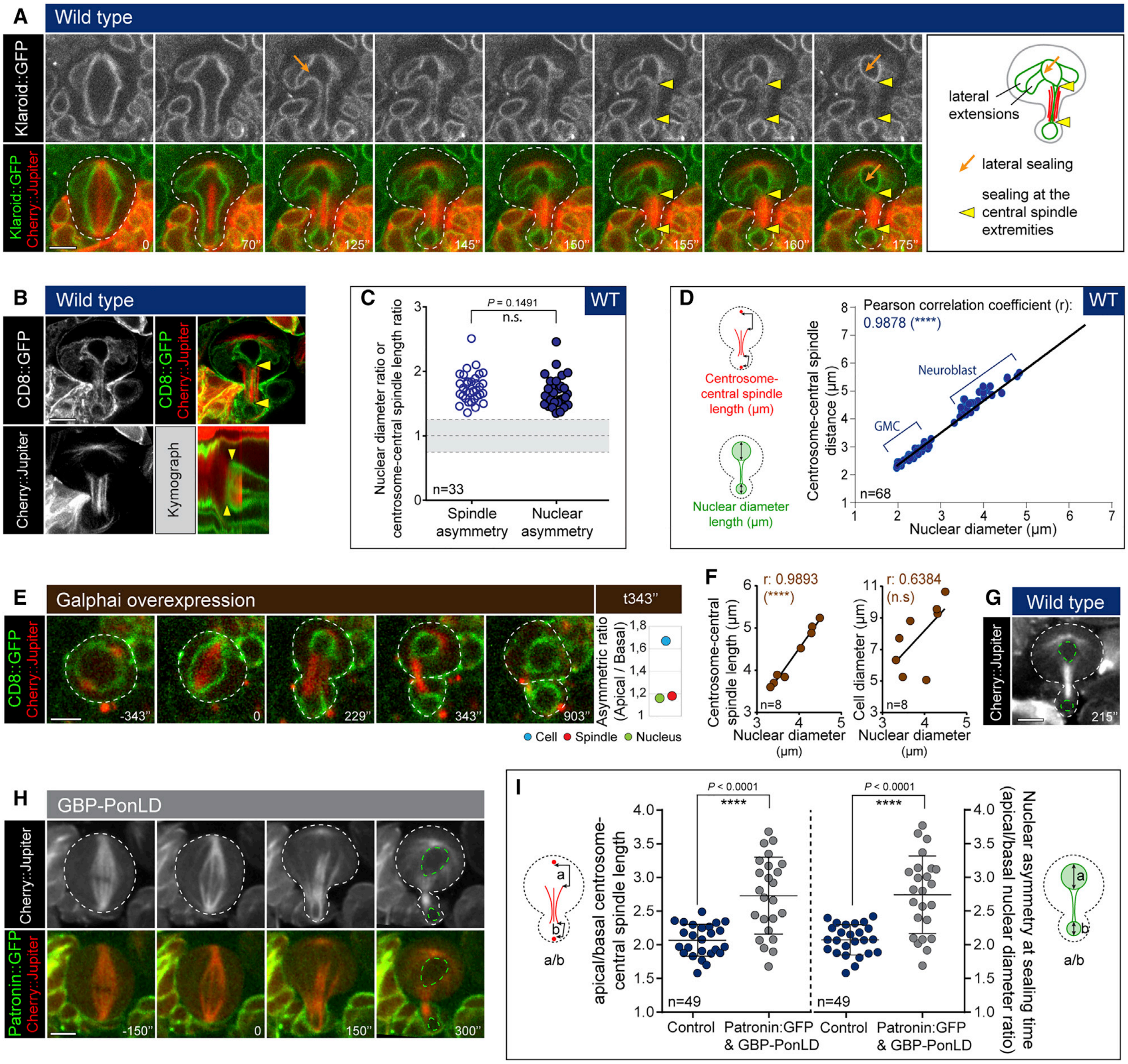

Figure 4. Asymmetric nuclear division depends on nuclear sealing at sites dictated by the spindle

(A) Dividing neuroblasts expressing Klaroid::GFP and Cherry::Jupiter. Orange and yellow arrows indicate envelope sealing events.

(B) Airyscan pictures of neuroblasts expressing CD8::GFP and Cherry::Jupiter. Yellow arrowheads show sealing sites. Kymographs are done along the apico-basal axis.

(C) Graph showing the spindle asymmetry ratio and the nuclear diameter ratio (n = 33). Asterisks denote statistical significance, derived from unpaired t tests: n.s., not significant.

(D) Plot showing correlation between spindle and nuclear asymmetries (n = 68).

(E) Representative time-lapse images of neuroblast expressing CD8::GFP, Cherry::Jupiter, and Galphai. Quantification shows the asymmetric ratio for the cell, nucleus, and spindle at the sealing time.

(F) Plot showing correlation between spindle and nuclear asymmetries (on the left) or between cell and nuclear asymmetries (on the right); (n = 8).

(G) Image of neuroblast at the nuclear sealing time, showing the asymmetry in the central spindle and nuclear size (green dashed line).

(H) Representative time-lapse images of neuroblast expressing Cherry::Jupiter, Patronin::GFP, and GBP-PonLD. Green dashed lines correspond to the nuclei.

(I) Graph showing the spindle asymmetry (left) and the nuclear asymmetry (right) for control neuroblasts and after expression of Patronin::GFP and GBP-PonLD (n = 25 and n = 24). Bars indicate mean ± standard deviation. Asterisks denote statistical significance, derived from unpaired t tests: ****$p \leq 0.0001$. For each experiment, $n \geq 3$ experiments.

Scale bars are 5 µm. See also Figure S4.

CellPress
OPEN ACCESS

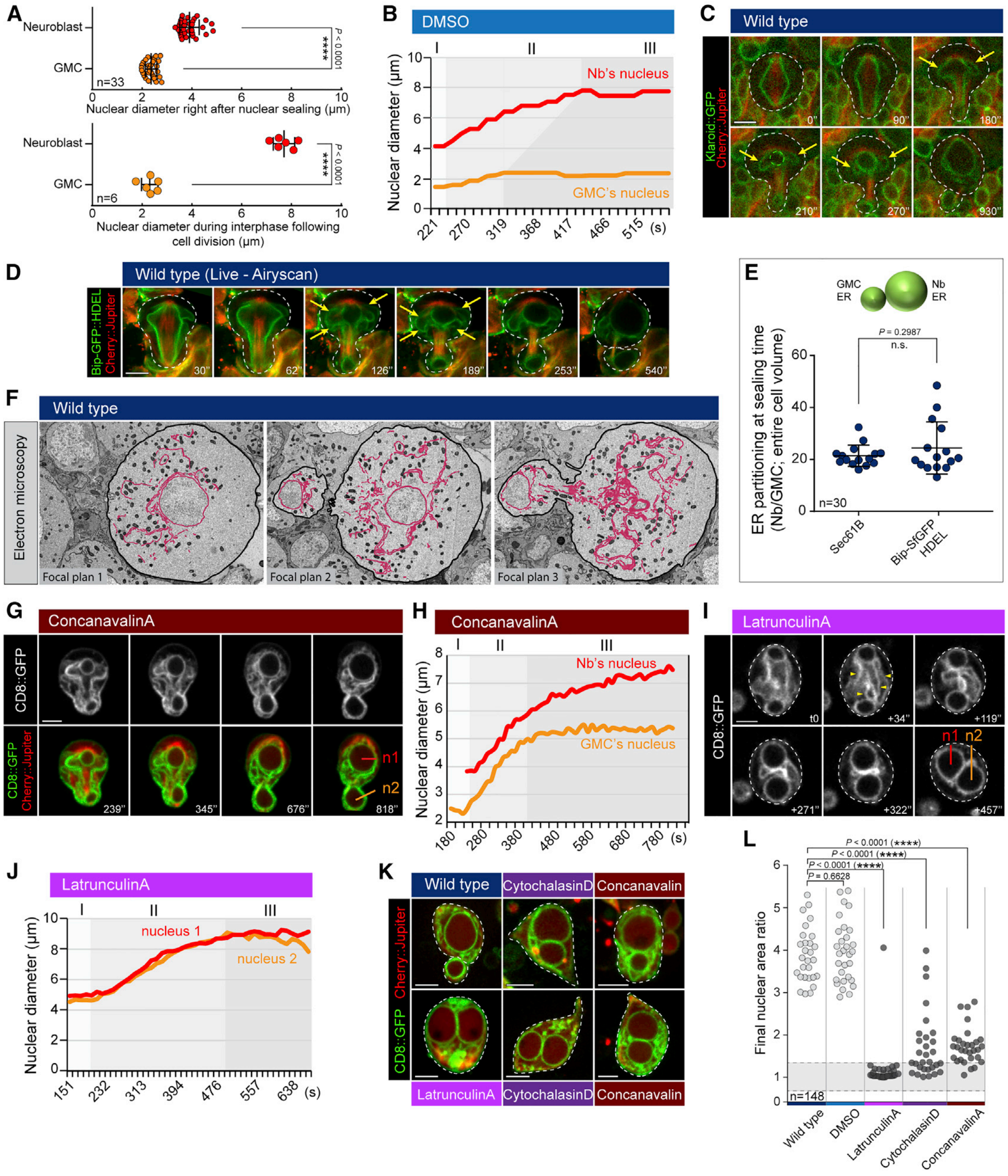

Figure 5. Final nuclear sizes are achieved via differential growth of the two daughter nuclei

(A) Scatterplots showing nuclear diameter of neuroblast and GMC right after nuclear sealing or during G1. Bars indicate mean ± standard deviation. Asterisks denote statistical significance, derived from unpaired t tests: ****p $\leq$ 0.0001.

(B) Graph showing the diameter of a neuroblast and GMC nucleus, from nuclear sealing until G1.

(C) Neuroblasts expressing Klaroid::GFP and Cherry::Jupiter; yellow arrows show cytoplasmic reservoir of nuclear membrane.

(D) Representative time-lapse images of neuroblast expressing the ER marker Bip-SfGFP::HDEL and Cherry::Jupiter, taken with Airyscan. Yellow arrows show cytoplasmic reservoir of nuclear membrane.

(E) Graph showing the asymmetric ER segregation at sealing time, using two different probes. The sum intensity is measured in the entire neuroblast and GMC volume, and the ratio Nb/GMC is plotted. Bars indicate mean ± standard deviation. Asterisks denote statistical significance, derived from unpaired t tests: n.s., not significant.

(F) Electron micrographs. ER/nuclear membranes are highlighted in pink.

(G) Representative time-lapse images of a neuroblast cultured on ConcanavalinA expressing CD8::GFP and Cherry::Jupiter.

(H) Graph corresponding to the neuroblast presented in (G), showing the size of both nuclei throughout the cell cycle.

(I) Representative time-lapse images of a Latrunculin-treated neuroblast expressing CD8::GFP. Yellow arrowheads indicate reservoir of available nuclear membrane.

(J) Graph from a representative Latrunculin-treated neuroblast showing the size of both nuclei throughout the cell cycle.

(legend continued on next page)

S5D; Video S7). Interestingly, the cytokinesis furrow appears to play a role in enforcing asymmetric segregation of this nuclear membrane reservoir, since both daughter nuclei grew with similar rates in cells that experienced a delay in cytokinesis as a result of having been plated on Concanavalin A-coated dishes[22] (Figure 5G, time points 239″ and 345″; Figure 5H, step II). Once the furrow narrowed to a point at which full access to bulk cytoplasmic ER was visibly restricted, growth of the GMC nucleus was inhibited, while the neuroblast nucleus continued to expand (Figure 5G, time point 818″; Figure 5H, step III). As a consequence, cells that failed division at very late stages of cytokinesis yielded binucleated cells containing one large and one small nucleus. By contrast, when Latrunculin A was used to prevent cleavage furrow initiation, both nuclei (which are born with an identical size as the result of a symmetrical spindle) grew at similar rates (Figures 5I and 5J, step II), before reaching a size plateau at the same time (step III) (Figures 3F, 5I, and 5J, step I). Thus, depending on spindle asymmetry, cleavage furrow dynamics, and the timing of cytokinesis failure, it is possible to generate binucleate cells with sibling nuclei that are similar or very different in size (Figures 5K and 5L). Based on these data, we propose a model in which differences in the final size of sibling nuclei depend on two processes that occur in sequence: (1) asymmetric nuclear sealing guided by asymmetries in the spindle and (2) differential nuclear growth based on ER/nuclear membrane availability.

Asymmetric nuclear division affects chromatin organization

As a result of these processes, nuclear division in neuroblasts generates daughter nuclei that differ profoundly in size despite their carrying the same amount of DNA, leading to an asymmetry in chromatin packing. This was first visible in anaphase/telophase, when the His2A signal was seen becoming weaker in the neuroblast nucleus as its genome decondensed to fill the larger nuclear compartment (Figures 6A, 6B, S6A, and S6B). This difference in packing was maintained after late cytokinesis failure (Figure S6C, left graph), in cells that displayed two nuclei that differed in size, but was abolished in binucleated cells containing two nuclei of similar size (Figure S6C, right graph).

The asymmetry in chromatin packing between the two nascent nuclei was accompanied by differences in H3K4me2 (a histone mark associated with neuronal differentiation in the mouse brain[25]), which arose during the process of nuclear division. The segregation of H3K4me2 was symmetric until late anaphase/early telophase (Figure 6C), but progression through telophase was associated with the development of a striking asymmetry in the distribution of H3K4me2 between the two daughter nuclei (Figures 6D and 6E, purple circles; from 1.057 ± 0.04 in anaphase/early telophase to 0.65 ± 0.05 in late telophase), which could not be explained by a different density due to the physical constraints of a small nuclear size (the control DAPI ratio remained around 1 throughout mitosis: Figure 6E, blue circles; 1.01 ± 0.06). As a result, following division, the H3K4me2 signal was high in GMC nuclei, but was barely detected in the neuroblast (Figure S6D). Furthermore, a similar asymmetry in H3K4me2 staining (0.77 ± 0.09, relative to 1.074 ± 0.12 for the DAPI channel) was observed in binucleated cells that possess a large and small nucleus within the same cytoplasm (Figure 6F). In line with these results, in control neuroblasts, the H3K4me2 signal was visibly asymmetric long before the cortical release and nuclear import of the cell fate determinant Prospero (Figure 6D, yellow channel; Figure 7A; 16.34 ± 2.34 min after anaphase onset). Taken together, these data reveal a role for asymmetric nuclear division alongside the polarized distribution of cell fate determinants in the generation of cell diversity in this system (Figure 7B).

DISCUSSION

Functional consequences of maintaining a nuclear compartment throughout mitosis

In this paper, by studying the nuclear envelope remodeling and partitioning during asymmetric divisions of *Drosophila* neural stem cells, we show that the nuclear compartment is not dissolved and reformed during passage through mitosis. Instead, a nuclear envelope persists, supported by an underlying mitotic lamina. Because NPCs are disassembled upon entry into mitosis, as nuclear membranes become fenestrated and multilayered (as reported for *C. elegans* embryos[11]), this envelope fails to function as a diffusion barrier. While, in an open mitosis, dispersed ER/nuclear membrane is recruited around the chromatin to re-establish the nuclear compartment anew as cells exit mitosis, in this system Lamin RNAi cells fail to reassemble their nuclei after division. Thus, fly neural stem cells appear to rely on the maintenance, remodeling, and inheritance of a nuclear compartment to generate daughter nuclei.

Forming two nuclei of different size

Importantly, while nuclear division in *Drosophila* neuroblasts is an autonomous process that is independent of cytokinesis, it is clear from our analysis that the asymmetry in nuclear division depends on the spindle and the position of the cytokinetic furrow. Asymmetric division is achieved in two steps. First, the asymmetric central spindle defines the sites at which the nuclear compartment is sealed, in a process that depends on Aurora B, Tumbleweed, and Pavarotti, leading to the formation of two nascent daughter nuclei that are moderately different in size. Second, as they grow, these nuclei appear to compete for the available reservoir of ER and nuclear membrane, as has been reported to be the case in sea urchin and *Xenopus* embryos.[26] Importantly, access to this membrane reservoir appears limited by partial closure of the cytokinesis furrow, since pinching of the plasma membrane was sufficient to limit nuclear growth

(K) Representative pictures showing wild-type and binucleated neuroblasts expressing CD8::GFP and Cherry::Jupiter, containing two nuclei with different or similar size.
(L) Scatterplot showing the nuclear area ratio between sibling nuclei for wild-type, DMSO-, Latrunculin-, Cytochalasin-, or Concanavalin-treated neuroblasts. Grey area on the graph represents a nuclear area ratio equal to 1 ± 0.25. Number of analyzed cells = 148. Asterisks denote statistical significance, derived from unpaired t tests: n.s., not significant, ****$p \leq 0.0001$. For each experiment, $n \geq 3$.
Scale bars are 5 μm. See also Figure S5 and Video S7.

CellPress
OPEN ACCESS

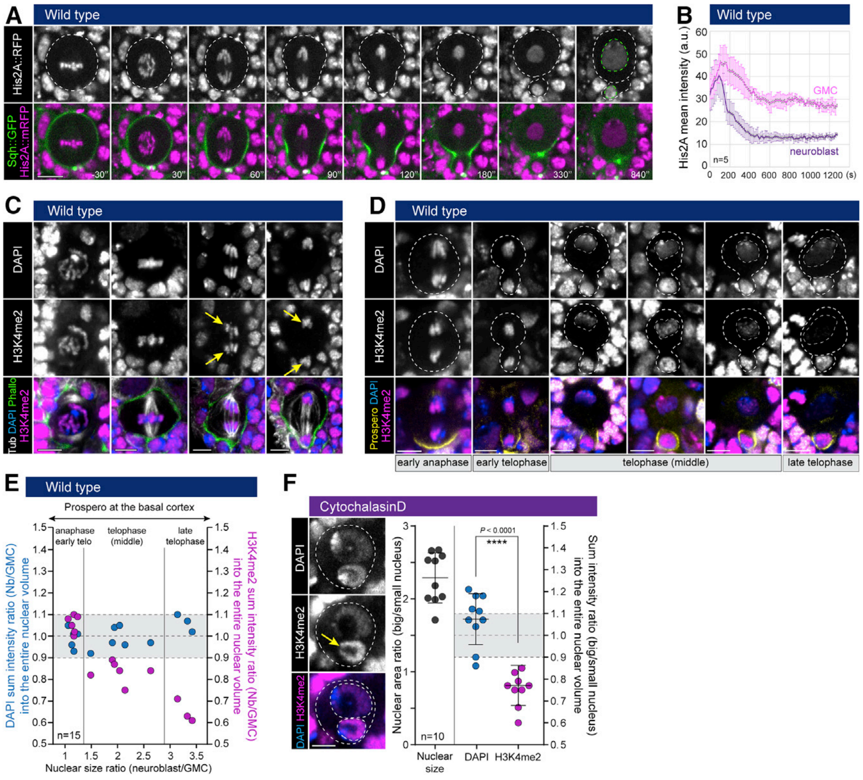

Figure 6. Asymmetric nuclear division affects chromatin organization

(A) Representative time-lapse images of a neuroblast expressing the DNA marker His2A::RFP and the cortical marker Sqh::GFP.

(B) Graph showing His2A::RFP mean intensity over time for GMC and neuroblast nuclei, n = 5. Error bars represent standard deviation.

(C) Wild-type neuroblasts fixed and stained for DAPI, H3K4me2, Tubulin, and Phalloidin. Yellow arrows indicate similar H3K4me2 intensity on the two pools of sister chromatids.

(D) Wild-type neuroblasts fixed and stained for DAPI, H3K4me2, and Prospero. White dashed lines show the nuclear area during and after nuclear growth.

(E) For cells from early anaphase to late telophase, graph (left) shows nuclear size ratio on the x axis versus ratio (neuroblast/GMC) of sum intensity for DAPI across the entire nuclear volume on the y axis, and (right) the equivalent for H3K4me2 on the y axis.

(F) Binucleated neuroblast stained for DAPI and H3K4me2. The graph shows the nuclear area ratio (left) and the DAPI or H3K4me2 sum intensity ratio (right) for binucleated neuroblasts displaying two nuclei with different size (n = 10). Bars indicate mean ± standard deviation. Asterisks denote statistical significance, derived from unpaired t tests: $^{**}p \le 0.01$. For each experiment, $n \ge 3$.

Scale bars are 5 μm. See also Figure S6.

before abscission, and since the early inhibition of furrow formation enabled the growth of the GMC nucleus. These data suggest that the cytokinesis furrow in fly neuroblasts forms a barrier that functions like the neck of dividing budding yeast cells[27,28] to restrict the movement of material between the two daughter cells prior to division. As a result, the asymmetry in nuclear size in this system is not the result of differences in cell volume, as has been suggested in other model systems.[29–32]

Functional implications of asymmetric nuclear division

As shown through our analysis, the process of asymmetric nuclear division generates two daughter nuclei that differ 37-fold

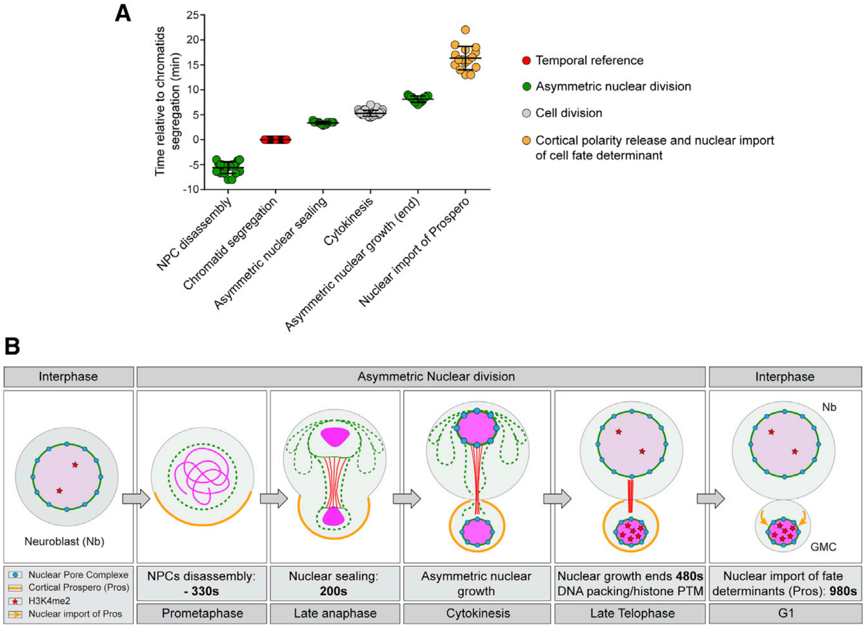

Figure 7. Asymmetric nuclear division generates two daughter nuclei that differ in size, envelope composition, and chromatin organization before the nuclear import of cell fate determinants

(A) Graph showing the timing of the successive steps related to nuclear division, cytokinesis, and nuclear import of Prospero.

(B) Diagram representing different steps of the process of asymmetric nuclear division and cell fate determinant release. NPCs disassemble 330 s before anaphase onset. The nuclear envelope is then asymmetrically sealed in a spindle-dependent manner 200 s after anaphase onset to generate two daughter nuclei with moderate differences in size. This asymmetry is increased by differential nuclear growth during telophase, until nuclear size stabilization 480 s after anaphase onset. This is associated with a differential packing of the chromatin and an asymmetric distribution of histone marks between the new neuroblast and GMC nuclei, before the nuclear import of cell fate determinants (980 s after anaphase onset).

in size. This large-scale physical asymmetry and the accompanying molecular asymmetries in nuclear envelope composition generated by the differential enrichment of NPCs in sibling nuclei (Figures 1D, 2A, and S1E) give rise to differences in the decompaction of chromatin in the two daughter nuclei, and are associated with asymmetries in H3K4me2 that precede the nuclear import of cell fate determinants like Prospero (Figures 6D, 7A, and 7B). Thus, these nuclear asymmetries are not the consequence of the action of cell fate determinants. Instead, they are likely to be factors that influence the ability of these transcription factors to execute their functions. This is in line with a recent study identifying a role for Ankle2, a nuclear envelope protein, in the asymmetric segregation of cell fate determinants in mitotic neural stems cells (as well as on nuclear remodeling and spindle positioning).[33]

One might ask why the system is set up in this way? While further work is required to answer this question, our analysis hints at this being a way to define nuclear identity and size very early in late telophase/earlyG1, which might be particularly important in cells like these which have a very short cell cycle (45 min to 1.30 h on average). Semi-closed mitosis may also aid asymmetric nuclear division itself. In addition, this mode of division may allow nascent nuclei to compete for common factors prior to cell division, facilitating the break in symmetry required to distinguish the neural stem cell from the more differentiated GMC.

Finally, while many of the consequences of this mechanism of semi-closed mitosis we describe here remain to be explored, our analysis suggests that asymmetric nuclear division might contribute, alongside cortical polarity, to the formation of sibling cells with different identity/fate.

STAR★METHODS

Detailed methods are provided in the online version of this paper and include the following:

- KEY RESOURCES TABLE
- RESOURCE AVAILABILITY
  - Lead Contact
  - Materials Availability
  - Data and Code Availability
- EXPERIMENTAL MODEL AND SUBJECT DETAILS
- METHOD DETAILS
  - Live imaging sample preparation
  - Primary neuroblast cultures
  - Antibodies and immunostaining
  - Small molecule inhibitors and chemical treatments
  - Electron microscopy
  - Confocal microscopy
- QUANTIFICATION AND STATISTICAL ANALYSIS
  - Image processing and calculations
  - Statistical analysis

SUPPLEMENTAL INFORMATION

Supplemental information can be found online at https://doi.org/10.1016/j.cub.2021.06.063.

ACKNOWLEDGMENTS

We thank Andrew Vaughan, John Gallagher, and Ki Hng for microscopy support; Talila Volk, Saverio Brogna, Christian Lehner, François Payre, and Emmanuel Derivery for reagents; Lucy Collinson and Matt Russell for help optimizing the EM protocol; and Marc Amoyel, Franck Pichaud, Yanlan Mao, Jens Januschke, Emmanuel Derivery, and the Baum lab for feedback on this manuscript. This work was supported by the MRC LMCB (MC_CF12266 grant), a Cancer Research UK program grant (C1529/A17343), and a BBSRC project grant (BB/R009732/1).

AUTHOR CONTRIBUTIONS

C.R. and B.B. conceived the project. C.R. performed and analyzed the experiments except the electron microscopy. I.J.W. performed the electron microscopy experiments. C.R. and B.B. wrote the manuscript.

DECLARATIONS OF INTERESTS

The authors declare no competing interests.

Received: October 28, 2020
Revised: May 12, 2021
Accepted: June 23, 2021
Published: July 22, 2021

REFERENCES

1. Margalit, A., Vlcek, S., Gruenbaum, Y., and Foisner, R. (2005). Breaking and making of the nuclear envelope. J. Cell. Biochem. *95*, 454–465.
2. Fields, A.P., and Thompson, L.J. (1995). The regulation of mitotic nuclear envelope breakdown: a role for multiple lamin kinases. Prog. Cell Cycle Res. *1*, 271–286.
3. Sazer, S., Lynch, M., and Needleman, D. (2014). Deciphering the evolutionary history of open and closed mitosis. Curr. Biol. *24*, R1099–R1103.
4. Samwer, M., Schneider, M.W.G., Hoefler, R., Schmalhorst, P.S., Jude, J.G., Zuber, J., and Gerlich, D.W. (2017). DNA Cross-Bridging Shapes a Single Nucleus from a Set of Mitotic Chromosomes. Cell *170*, 956–972.e23.
5. Kutay, U., and Hetzer, M.W. (2008). Reorganization of the nuclear envelope during open mitosis. Curr. Opin. Cell Biol. *20*, 669–677.
6. Kubai, D.F., and Ris, H. (1969). Division in the dinoflagellate Gyrodinium cohnii (Schiller). A new type of nuclear reproduction. J. Cell Biol. *40*, 508–528.
7. Expósito-Serrano, M., Sánchez-Molina, A., Gallardo, P., Salas-Pino, S., and Daga, R.R. (2020). Selective Nuclear Pore Complex Removal Drives Nuclear Envelope Division in Fission Yeast. Curr. Biol. *30*, 3212–3222.e2.
8. Dey, G., Culley, S., Curran, S., Schmidt, U., Henriques, R., Kukulski, W., and Baum, B. (2020). Closed mitosis requires local disassembly of the nuclear envelope. Nature *585*, 119–123.
9. Katsani, K.R., Karess, R.E., Dostatni, N., and Doye, V. (2008). In vivo dynamics of Drosophila nuclear envelope components. Mol. Biol. Cell *19*, 3652–3666.
10. Lee, K.K., Gruenbaum, Y., Spann, P., Liu, J., and Wilson, K.L. (2000). C. elegans nuclear envelope proteins emerin, MAN1, lamin, and nucleoporins reveal unique timing of nuclear envelope breakdown during mitosis. Mol. Biol. Cell *11*, 3089–3099.
11. Rahman, M., Chang, I.Y., Harned, A., Maheshwari, R., Amoateng, K., Narayan, K., and Cohen-Fix, O. (2020). C. elegans pronuclei fuse after fertilization through a novel membrane structure. J. Cell Biol. *219*, 219.
12. Strunov, A., Boldyreva, L.V., Andreyeva, E.N., Pavlova, G.A., Popova, J.V., Razuvaeva, A.V., Anders, A.F., Renda, F., Pindyurin, A.V., Gatti, M., and Kiseleva, E. (2018). Ultrastructural analysis of mitotic Drosophila S2 cells identifies distinctive microtubule and intracellular membrane behaviors. BMC Biol. *16*, 68.

CellPress
OPEN ACCESS

13. De Souza, C.P., and Osmani, S.A. (2007). Mitosis, not just open or closed. Eukaryot. Cell *6*, 1521–1527.
14. Stafstrom, J.P., and Staehelin, L.A. (1984). Dynamics of the nuclear envelope and of nuclear pore complexes during mitosis in the Drosophila embryo. Eur. J. Cell Biol. *34*, 179–189.
15. Roubinet, C., Tsankova, A., Pham, T.T., Monnard, A., Caussinus, E., Affolter, M., and Cabernard, C. (2017). Spatio-temporally separated cortical flows and spindle geometry establish physical asymmetry in fly neural stem cells. Nat. Commun. *8*, 1383.
16. Roth, M., Roubinet, C., Iffländer, N., Ferrand, A., and Cabernard, C. (2015). Asymmetrically dividing Drosophila neuroblasts utilize two spatially and temporally independent cytokinesis pathways. Nat. Commun. *6*, 6551.
17. Roubinet, C., and Cabernard, C. (2014). Control of asymmetric cell division. Curr. Opin. Cell Biol. *31*, 84–91.
18. Homem, C.C., and Knoblich, J.A. (2012). Drosophila neuroblasts: a model for stem cell biology. Development *139*, 4297–4310.
19. Loyer, N., and Januschke, J. (2020). Where does asymmetry come from? Illustrating principles of polarity and asymmetry establishment in Drosophila neuroblasts. Curr. Opin. Cell Biol. *62*, 70–77.
20. Lee, T., and Luo, L. (1999). Mosaic analysis with a repressible cell marker for studies of gene function in neuronal morphogenesis. Neuron *22*, 451–461.
21. Mehsen, H., Boudreau, V., Garrido, D., Bourouh, M., Larouche, M., Maddox, P.S., Swan, A., and Archambault, V. (2018). PP2A-B55 promotes nuclear envelope reformation after mitosis in *Drosophila*. J. Cell Biol. *217*, 4106–4123.
22. Lamb-Wharton, R.J., and Morgan, W.T. (1993). Induction of T-lymphocyte adhesion by histidine-proline-rich glycoprotein and concanavalin A. Cell. Immunol. *152*, 544–555.
23. Smurnyy, Y., Toms, A.V., Hickson, G.R., Eck, M.J., and Eggert, U.S. (2010). Binucleine 2, an isoform-specific inhibitor of Drosophila Aurora B kinase, provides insights into the mechanism of cytokinesis. ACS Chem. Biol. *5*, 1015–1020.
24. Schaefer, M., Petronczki, M., Dorner, D., Forte, M., and Knoblich, J.A. (2001). Heterotrimeric G proteins direct two modes of asymmetric cell division in the Drosophila nervous system. Cell *107*, 183–194.
25. Zhang, J., Parvin, J., and Huang, K. (2012). Redistribution of H3K4me2 on neural tissue specific genes during mouse brain development. BMC Genomics *13* (*Suppl 8*), S5.
26. Mukherjee, R.N., Sallé, J., Dmitrieff, S., Nelson, K.M., Oakey, J., Minc, N., and Levy, D.L. (2020). The Perinuclear ER Scales Nuclear Size Independently of Cell Size in Early Embryos. Dev. Cell *54*, 395–409.e7.
27. Shcheprova, Z., Baldi, S., Frei, S.B., Gonnet, G., and Barral, Y. (2008). A mechanism for asymmetric segregation of age during yeast budding. Nature *454*, 728–734.
28. Boettcher, B., Marquez-Lago, T.T., Bayer, M., Weiss, E.L., and Barral, Y. (2012). Nuclear envelope morphology constrains diffusion and promotes asymmetric protein segregation in closed mitosis. J. Cell Biol. *197*, 921–937.
29. Cantwell, H., and Nurse, P. (2019). Unravelling nuclear size control. Curr. Genet. *65*, 1281–1285.
30. Mukherjee, R.N., Chen, P., and Levy, D.L. (2016). Recent advances in understanding nuclear size and shape. Nucleus *7*, 167–186.
31. Chan, Y.H., and Marshall, W.F. (2010). Scaling properties of cell and organelle size. Organogenesis *6*, 88–96.
32. Webster, M., Witkin, K.L., and Cohen-Fix, O. (2009). Sizing up the nucleus: nuclear shape, size and nuclear-envelope assembly. J. Cell Sci. *122*, 1477–1486.
33. Link, N., Chung, H., Jolly, A., Withers, M., Tepe, B., Arenkiel, B.R., Shah, P.S., Krogan, N.J., Aydin, H., Geckinli, B.B., et al. (2019). Mutations in ANKLE2, a ZIKA Virus Target, Disrupt an Asymmetric Cell Division Pathway in Drosophila Neuroblasts to Cause Microcephaly. Dev. Cell *51*, 713–729.e6.
34. Somers, W.G., and Saint, R. (2003). A RhoGEF and Rho family GTPase-activating protein complex links the contractile ring to cortical microtubules at the onset of cytokinesis. Dev. Cell *4*, 29–39.
35. Chan, L.N., and Gehring, W. (1971). Determination of blastoderm cells in Drosophila melanogaster. Proc. Natl. Acad. Sci. USA *68*, 2217–2221.
36. Elhanany-Tamir, H., Yu, Y.V., Shnayder, M., Jain, A., Welte, M., and Volk, T. (2012). Organelle positioning in muscles requires cooperation between two KASH proteins and microtubules. J. Cell Biol. *198*, 833–846.
37. Rugjee, K.N., Roy Chaudhury, S., Al-Jubran, K., Ramanathan, P., Matina, T., Wen, J., and Brogna, S. (2013). Fluorescent protein tagging confirms the presence of ribosomal proteins at Drosophila polytene chromosomes. PeerJ *1*, e15.
38. Radermacher, P.T., Myachina, F., Bosshardt, F., Pandey, R., Mariappa, D., Müller, H.A., and Lehner, C.F. (2014). O-GlcNAc reports ambient temperature and confers heat resistance on ectotherm development. Proc. Natl. Acad. Sci. USA *111*, 5592–5597.
39. Buszczak, M., Paterno, S., Lighthouse, D., Bachman, J., Planck, J., Owen, S., Skora, A.D., Nystul, T.G., Ohlstein, B., Allen, A., et al. (2007). The carnegie protein trap library: a versatile tool for Drosophila developmental studies. Genetics *175*, 1505–1531.
40. Chabu, C., and Doe, C.Q. (2008). Dap160/intersectin binds and activates aPKC to regulate cell polarity and cell cycle progression. Development *135*, 2739–2746.
41. Schneider, C.A., Rasband, W.S., and Eliceiri, K.W. (2012). NIH Image to ImageJ: 25 years of image analysis. Nat. Methods *9*, 671–675.
42. Summerville, J.B., Faust, J.F., Fan, E., Pendin, D., Daga, A., Formella, J., Stern, M., and McNew, J.A. (2016). The effects of ER morphology on synaptic structure and function in Drosophila melanogaster. J. Cell Sci. *129*, 1635–1648.
43. Derivery, E., Seum, C., Daeden, A., Loubéry, S., Holtzer, L., Jülicher, F., and Gonzalez-Gaitan, M. (2015). Polarized endosome dynamics by spindle asymmetry during asymmetric cell division. Nature *528*, 280–285.
44. Dietzl, G., Chen, D., Schnorrer, F., Su, K.C., Barinova, Y., Fellner, M., Gasser, B., Kinsey, K., Oppel, S., Scheiblauer, S., et al. (2007). A genome-wide transgenic RNAi library for conditional gene inactivation in Drosophila. Nature *448*, 151–156.
45. Albertson, R., and Doe, C.Q. (2003). Dlg, Scrib and Lgl regulate neuroblast cell size and mitotic spindle asymmetry. Nat. Cell Biol. *5*, 166–170.

STAR★METHODS

KEY RESOURCES TABLE

REAGENT or RESOURCE	SOURCE	IDENTIFIER
**Antibodies**		
Mouse anti-LamDm0 (1:100)	DHSB	Cat# ADL67.10; RRID:AB_528336
LamDm0 Ser25 (1:100)	DHSB	Cat# ADL84.12; RRID:AB_528338
Rabbit anti-dMoesin (1:1000)	François Payre lab	N/A
Chicken anti-GFP (1:1000)	Abcam	Cat# ab13970; RRID:AB_300798
Rabbit anti-H3K4me2 (1:100)	Abcam	Cat# ab7766; RRID:AB_2560996
Mouse anti-Prospero (1:100)	DHSB	Cat# MR1A; RRID:AB_528440
Vectashield with DAPI	Vector Laboratories	Cat# H1200; RRID:AB_2336790
**Biological Samples**		
Larval brain tissues from *Drosophila melanogaster* strains	This study	N/A
**Chemicals, Peptides, and Recombinant Proteins**		
Schneider's Insect Medium	Sigma-Aldrich	Cat# S0146
Insulin	Sigma-Aldrich	Cat# I6634
L-Glutamine	Sigma-Aldrich	Cat# G7513
L-Glutathione reduced	Sigma-Aldrich	Cat# G6529
20-Hydroxyecdysone	Sigma-Aldrich	Cat# H5142
FBS	Sigma-Aldrich	Cat# F6178
Chan and Gehring's medium	[35]	N/A
Papain	Sigma-Aldrich	Cat# P4762
Collagenase Type I	Sigma-Aldrich	Cat# C2674
Concanavalin A	Sigma-Aldrich	Cat# L7647
Cytochalasin D	Sigma-Aldrich	Cat# C8273
Latrunculin A	Sigma-Aldrich	Cat# L5163
Binuclein 2	Sigma-Aldrich	Cat# B1186
MitoTracker Deep Red	Invitrogen	Cat# M22426
**Experimental Models: Organisms/Strains**		
D.melanogaster: y, w; Klaroid^{CB04483}	Bloomington Drosophila Stock Center	BDSC: 51525 FBal0211641
D.melanogaster: w; UAS BiP::sfGFP::HDEL	Bloomington Drosophila Stock Center	BDSC: 64749 FBst0064749
D.melanogaster: UAS Klarsicht::GFP	Elhanany-Tamir et al.[36]	N/A
D.melanogaster: UAS RpS13::GFP	Rugjee et al.[37]	FBal0284510
D.melanogaster: gNup58::EGFP	Radermacher et al.[38]	FBtp0093711
D.melanogaster: w; EGFP::Nup107	Bloomington Drosophila Stock Center	BDSC: 35514 FBst0035514
D.melanogaster: yw; UAS LamDm0::GFP	Bloomington Drosophila Stock Center; Buszczak et al.[39]	BDSC: 7376 FBst0007376
D.melanogaster: w; WorGal4, UAS Cherry::Jupiter / Cyo; UAS CD8::GFP / TM6B,Tb	This study	N/A
D.melanogaster: UAS EGFP::NLS(tra)	Bloomington Drosophila Stock Center	BDSC: 65402 FBst0065402
D.melanogaster: UAS Pebble::GFP	Somers et al. [34]	N/A
D.melanogaster: w; WorGal4, Klaroid^{CB04483}, UAS Cherry::Jupiter / Cyo; UAS Dicer / TM6B,Tb	This study	N/A
D.melanogaster: y,sc,v,sev; +; UAS dsRNA LamDm0	Bloomington Drosophila Stock Center	BDSC: 36617 FBst0036617

(*Continued on next page*)

Continued

REAGENT or RESOURCE	SOURCE	IDENTIFIER
D.melanogaster: y, sc, v, sev; UAS dsRNA LamDm0; +	Bloomington Drosophila Stock Center	BDSC: 57501 FBst0057501
D.melanogaster: UAS Cherry::Jupiter	Chabu and Doe[40]	FBal0220463
D.melanogaster: w;WorGal4, His2A::mRFP / Cyo; MKRS / TM6B,Tb	Roubinet et al.[15]	N/A
Software and Algorithms		
ImageJ	Schneider et al.[41]	https://imagej.nih.gov/ij/index.html; RRID:SCR_003070
Imaris	Bitplane	https://imaris.oxinst.com/packages; RRID:SCR_007370
Volocity	PerkinElmer	http://quorumtechnologies.com/index.php/component/content/category/31-volocity-software; RRID:SCR_002668
NIS-Element	Nikon	https://www.microscope.healthcare.nikon.com/products/software; RRID:SCR_014329
Slidebook	3i	https://www.intelligent-imaging.com/slidebook; RRID:SCR_014300
Prism	GraphPad	https://www.graphpad.com/; RRID:SCR_002798
Photoshop	Adobe	https://www.adobe.com/uk/products/photoshop.html; RRID:SCR_014199
Illustrator	Adobe	https://www.adobe.com/uk/products/illustrator.html; RRID:SCR_010279

RESOURCE AVAILABILITY

Lead Contact

Further information and requests for resources and reagents should be directed to and will be fulfilled by the Lead Contact, Chantal Roubinet (c.roubinet@ucl.ac.uk).

Materials Availability

All unique/stable reagents generated in this study are available from the Lead Contact without restriction.

Data and Code Availability

- All data reported in this paper will be shared by the lead contact upon request.
- This paper does not report original code.
- Any additional information required to reanalyze the data reported in this paper is available from the lead contact upon request.

EXPERIMENTAL MODEL AND SUBJECT DETAILS

Several lines of the fruit fly *Drosophila melanogaster* were used in this study. Flies and larvae were raised in vials containing standard cornmeal-agar medium supplemented with baker's yeast and incubated at 25°C. Third-instar larvae of 3-days old (expressing live imaging markers) or 5-days old (expressing RNAi) were dissected to extract the brains, then live imaging was performed directly on brains or after brain dissociation (see "Method details"). Experiments utilized both female and male larvae, and data from both sexes was pooled.

Flies used in this study include: Klaroid^{CB04483} (BDSC-51525),[39] UAS BiP::sfGFP::HDEL (BDSC-64749),[42] UAS Klarsicht::GFP,[36] UAS RpS13::GFP,[37] gNup58::EGFP,[38] EGFP::Nup107 (BDSC-35514),[9] UAS LamDm0::GFP (BDSC-7376), UAS 5EGFP::NLS (BDSC-65402), UAS dsRNA LamDm0 (BDSC-36617 and BDSC-57501), WorGal4, UAS Cherry::Jupiter; UAS CD8::GFP (this study), WorGal4, Klaroid^{CB04483}, UAS Cherry::Jupiter; UAS Dicer (this study), UAS Galphai (BDSC-44600), w; UAS GBP::PonLD; UAS GFP::Patronin,[43] UAS AuroraB RNAi (VDRC-35107),[44] UAS AuroraB RNAi (BDSC-35299),[44] w; WorGal4, UAS His2A::mRFP,[15] UAS Tumbleweed RNAi (BDSC-28982 and VDRC-106850), UAS Pavarotti RNAi (BDSC-35649), w; WorGal4, His2A::mRFP, Sqh::GFP.[15] Transgenes were expressed using the neuroblast-specific driver worGal4.[45] Detailed allele information is listed in the Key resources table.

METHOD DETAILS

Live imaging sample preparation

For experiments performed on intact brains, imaging medium (Schneider's insect medium mixed with 10% FBS (Sigma), 2% Pen-StrepNeo (Sigma), 0.02 mg/mL insulin (Sigma), 20mM L-glutamine (Sigma), 0.04 mg/mL L-glutathione (Sigma) and 5 μg/mL 20-hydroxyecdysone (Sigma)) was warmed up to room temperature before use. Seventy-two hours after egg laying, larvae were dissected in imaging medium then brains were transferred onto a gas-permeable membrane (YSI Life Sciences #13-298-83) fitted on a metallic slide. Brains were oriented with the brain lobes facing the coverslip, and excess media was removed until the brain lobes were in contact with the coverslip. The sample was sealed with Vaseline.

Primary neuroblast cultures

For experiments done on primary neuroblast culture, seventy-two hour larvae were dissected in Chang & Gerhing solution (3.2 g/L NaCl, 3 g/L KCL, 0.69 g/L CaCl2-2H2O, 3.7 g/L MgSO4-7H2O, 1.79 g/L tricine buffer pH 7, 3.6 g/L glucose, 17.1 g/L sucrose, 1 g/L BSA)[35] at room temperature. Brains were then incubated in Chang & Gerhing solution supplemented in collagenase from Clostridium histolyticum (Sigma) and papain from papaya latex (Sigma) at a final concentration of 1 mg/mL each, during 45 min at 29°C. Brains were washed with imaging medium (see above) then dissociated in imaging medium by pipetting 20–25 times. The cell culture was then imaged using Ibidi chambers (15μ-slide angiogenesis).

Antibodies and immunostaining

The following primary antibodies were used for this study: mouse anti-LamDm0 Ser25 (DHSB; #ADL84.12; 1:100), mouse anti-LamDm0 (DHSB; #ADL67.10; 1:100), chicken anti-GFP (Abcam; #ab13970; 1:1000), rabbit anti-dMoesin (gift from Francois Payre; 1:1000), rabbit anti-H3K4me2 (Abcam; #ab7766; 1;100) and rabbit anti-Miranda (gift from Emmanuel Caussinus; 1:50). Secondary antibodies were from Invitrogen and Phalloidin-FITC (Sigma; #P5282; 1:100) was used to stain F-Actin.

For immunostaining, seventy-two hour larvae were dissected in Schneider's insect medium (Sigma-Aldrich S0146) and brains were fixed for 20 min in 4% paraformaldehyde in PEM (100 mM PIPES pH 6.9, 1 mM EGTA and 1 mM MgSO4). After fixing, the brains were washed with PBSBT (1 × PBS (pH7,4), 0.1% Triton X-100 and 1% BSA) then blocked with PBSBT for 1 h. Primary antibody dilution was prepared in PBSBT and brains were incubated for 48 hours at 4°C. Brains were washed with PBSBT four times for 30 min each, then incubated with secondary antibodies diluted in PBSBT at 4°C overnight. The next day, brains were washed with PBST (1x PBS, 0.1% Triton X-100) four times for 20 min each and kept in Vectashield antifade mounting medium with DAPI (Vector laboratories; H1200), at 4°C.

Small molecule inhibitors and chemical treatments

Following dissection and prior montage for live imaging, brains were incubated in imaging medium (see above) containing the following chemical reagents, as indicated on the Figures: Latrunculin A (Sigma; #L5163; final concentration of 10 μM), Cytochalasin D (Sigma; #C8273; final concentration of 5 μg/mL or 10 μg/mL) or Binuclein 2 (Sigma; #B1186; final concentration of 10 μM or 20 μM). Cerulenin (Sigma; #C2389; 10μM) was added during live imaging. After brain dissociation, the following final concentrations were used on primary neuroblast culture: Latrunculin A: 1 μM; Cytochalasin D: 0.5 μg/mL.

For control experiments, dissected brains were incubated in DMSO at the same final concentrations than the ones used to dissolve the reagents.

In order to induce binucleated cells, primary neuroblast culture was platted on dish coated with Concanavalin A (Sigma # L7647).

Observation of mitochondria distribution during mitosis was done by incubating primary neuroblast culture with MitoTracker Deep Red (Invitrogen # M22426; final concentration of 0.1nM).

Electron microscopy

Cells were cultured on gridded coverslip-bottomed dishes (MatTek, P35G-1.5–14-CGRD) to facilitate correlation between light and electron microscopy, and processed for the latter following a protocol adapted from Deerinck et al. (NCMIR methods for 3D EM: A new protocol for preparation of biological specimens for serial block face scanning electron microscopy). Briefly, coverslips were fixed in 2% PFA / 2.5% Gluteraldehyde solution (EM grade, TAAB) in 0.1M sodium cacodylate buffer for 30 min at room temperature, washed in 0.1M sodium cacodylate buffer and post-fixed in 1% OsO_4/1.5% potassium ferricyanide for 1 h at 4°C. Samples were then treated with 1% thiocarbohydrazide (TCH) for 20 min at room temperature, 2% OsO_4 for 30 min at room temperature, 1% uranyl acetate (UA) overnight at 4°C and lead aspartate for 30 min at 60°C, with washing in dH_2O between each step. This was followed by dehydration of the samples by graded ethanol incubations in 70%, 90% and 100% ethanol, and incubations for 1 hour in a 1:1 mix of propylene oxide: epon resin (TAAB), 1 hour in fresh 100% epon resin, and a change to a final 1 hour epon resin incubation all at room temperature before mounting the coverslip by inversion onto a pre-polymerized epon resin stub and polymerization overnight at 60°C. Coverslips were removed by plunging into liquid nitrogen. Cells of interest were relocated on the blockface using the transferred alphanumeric grid with reference to light microscopy images, and the resin block trimmed accordingly. Ultrathin sections of 70 nm were cut using a diamond ultra 45-degree knife (Diatome) on a Leica UC7 ultramicrotome, and collected on formvar coated copper 2 × 1mm slot grids. EM images were acquired on a transmission electron microscope (T12 Tecnai Spirit biotwin, FEI).

Confocal microscopy

Fixed samples were imaged using an inverted Leica SP5 confocal microscope. For representative images, a 60 × /1.40 N.A oil immersion objective was used. Live cell imaging was performed on a UltraView Vox spinning disc confocal microscope (Perkin Elmer Nikon TiE; Yokogawa CSU-X1 spinning disc scan head) with 60 × /1.40 N.A oil objective and equipped with a Hamamatsu C9100-13 EMCCD camera, or a 3I spinning disc confocal microscope (Zeiss AxioObserver Z1; Yokogawa CSU-W1 spinning disc scan head) with 63 × /1.40 N.A objective and equipped with a photometrics prime 95B scientific CMOS camera. Both spinning disc microscopes are equipped with a temperature-controlled environment chamber set at 26C for the experiments. Airyscan pictures were taken on fixed and stained neuroblasts using an inverted LSM880 Multiphoton or on live samples using a LSM900 point scanning laser confocal with Airyscan 2 on an inverted Axio Observer Z1 microscope stand.

QUANTIFICATION AND STATISTICAL ANALYSIS

Image processing and calculations

Images were processed using Imaris × 64 7.5.2 and ImageJ softwares.

Measurements of intensity was obtained by using the oblique slicer in Imaris oriented along the cell division axis and going through the mid-plan of both daughter nuclei (neuroblast and GMC, respectively). The corresponding images were exported as TIFF files and opened with ImageJ to measure the mean intensity or integrated density, as mentioned on graphs. To measure the ratio cytoplasmic / nuclear NLS::5GFP or Rps13::GFP, background correction was performed by measuring the green channel intensity in the media. For the cell width and nuclear width values over time, a grid was used to measure the cell and nuclear width along each of the line of the grid, starting from the apical cortex toward the basal one for each time point. Cell and nuclear furrowing values were obtained by measuring over time both cell and nuclear width along the red line, which represents the cleavage furrow plan. To do so, images were exported as TIFFs then combined in a stack using ImageJ. The red line was drawn at anaphase when the cleavage furrow positioning is set and easy to determine. Measurements of both compartment widths were then done along this line, prior and after anaphase onset, to quantify the cleavage furrow ingression and the deformation of the nuclear compartment at the cleavage furrow region. Measurements of nuclear or cell area ratio were performed by using oblique slicer in Imaris as previously described, then images were exported as TIFFs and opened with ImageJ to measure manually the area or perimeter (as mentioned on the figures) by drawing the border of each nucleus and cell. For the graphs showing nuclear growth, TIFF files were opened with ImageJ then the nuclear diameter was manually measured for each time point. Microtubule density was based on the measurement of Cherry::Jupiter intensity, as reported in Derivery et al.[43] For the experiments with nanobody (GBP-PonLD), the nuclear size was measured using the Patronin channel, since Patronin is also present in the cytoplasm and excluded from the nuclei. ER partitioning between the daughter neuroblast and daughter GMC was measured using two different ER probes, measuring the sum intensity into the entire volume of the nuclei. H3K4me2 measurements were done by quantifying the sum intensity of this Histone mark in the entire volume of the two nuclei for each binucleated cell, plotting the ratio on the graph. All figures were assembled using Adobe Illustrator CS6 software.

Statistical analysis

For each experiment, the data was collected from at least 3 independent experiments. For each independent experiment, at least 8 larvae were dissected. For all the analysis, “n” refers to the number of cells analyzed and is mentioned on each graph as well as in figure legends. Statistical significance was determined with Student’s t test using GraphPad Prism software. In all Figures the Prism convention is used: n.s. ($p > 0.05$), *($P \leq 0.05$), **($P \leq 0.01$), ***($P \leq 0.001$) and ****($P \leq 0.0001$). In all graphs showing mean, the error bars correspond to standard deviation.

Current Biology, Volume *31*

Supplemental Information

Asymmetric nuclear division in neural stem cells generates sibling nuclei that differ in size, envelope composition, and chromatin organization

Chantal Roubinet, Ian J. White, and Buzz Baum

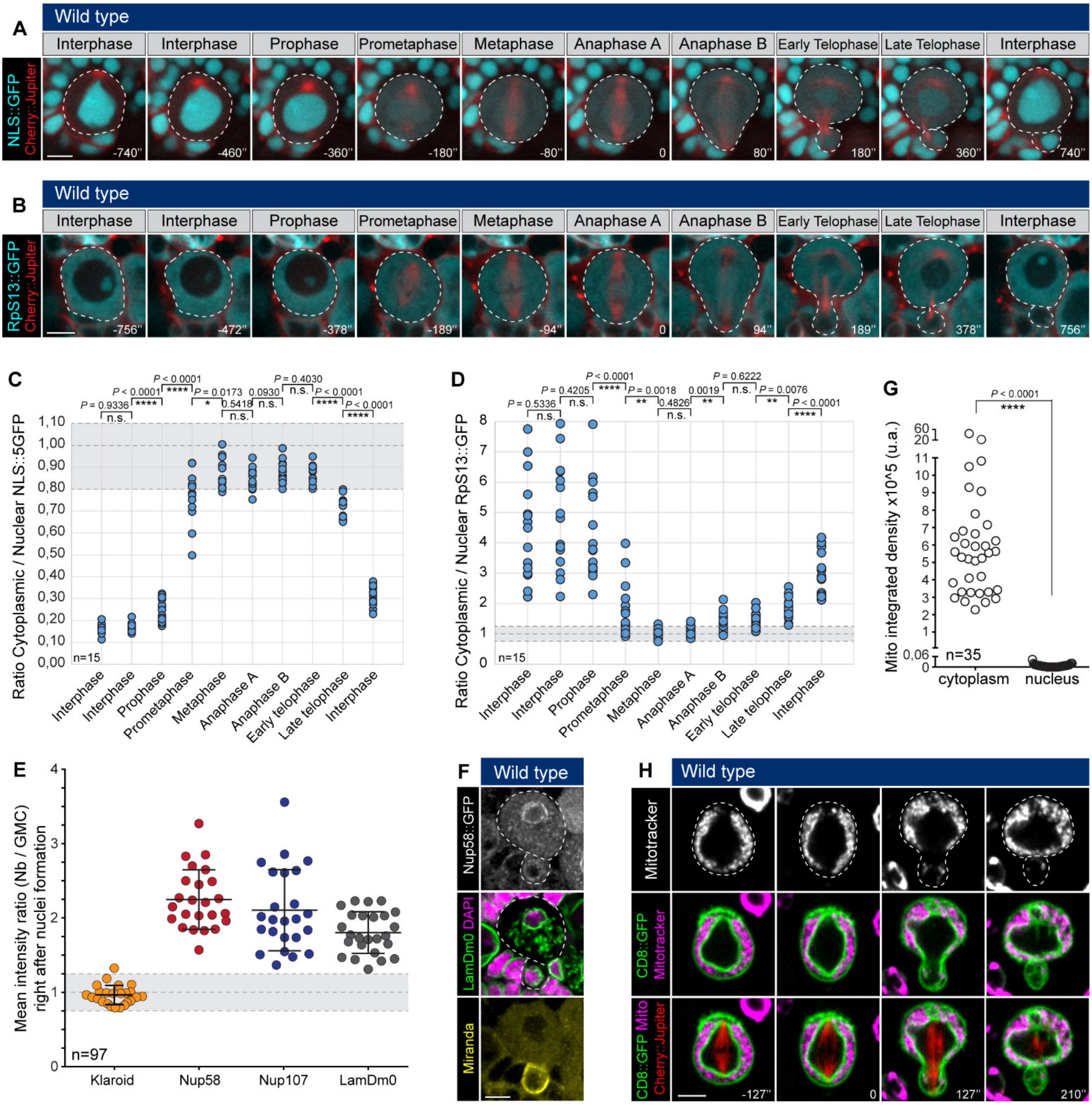

A
Wild type
Interphase
Interphase
Prophase
Prometaphase
Metaphase
Anaphase A
Anaphase B
Early Telophase
Late Telophase
Interphase
NLS::GFP
Cherry::Jupiter
-740''
-460''
-360''
-180''
-80''
0
80''
180''
360''
740''
B
Wild type
RpS13::GFP
Cherry::Jupiter
-756''
-472''
-378''
-189''
-94''
0
94''
189''
378''
756''
C
Ratio Cytoplasmic / Nuclear NLS::5GFP
n=15
D
Ratio Cytoplasmic / Nuclear RpS13::GFP
n=15
G
Mito integrated density x10^5 (u.a.)
n=35
cytoplasm
nucleus
P < 0.0001
E
Mean intensity ratio (Nb / GMC) right after nuclei formation
n=97
Klaroid
Nup58
Nup107
LamDm0
F
Wild type
Nup58::GFP
LamDm0 DAPI
Miranda
H
Wild type
Mitotracker
CD8::GFP Mitotracker
CD8::GFP Mito Cherry::Jupiter
-127''
0
127''
210''

Figure S1: The nuclear envelope of fly neuroblasts is maintained during mitosis and remodelled to generate two sibling nuclei that differ in size and composition. Related to Figure 1.

(**A-B**), Time lapse of dividing neuroblast expressing Cherry::Jupiter (red) and (**A**) NLS::5GFP (blue) or (**B**) RpS13::GFP (blue). (**C-D**) Mean intensity of (**C**) NLS::5GFP or (**D**) RpS13::GFP is measured in the cytoplasm and in the nuclear compartment for 10 time points throughout the cell cycle, then the ratio (cytoplasm/nuclear compartment) is plotted on the graph. The grey area corresponds to a ratio equal to 1 ± 0.25. Number of analysed cells = 15 for each condition. Asterisks denote statistical significance, derived from unpaired t tests: n.s.: not significant, *: $p \leq 0.05$, **: $p \leq 0.01$ and ****: $p \leq 0.0001$. (**E**) Graph showing the mean intensity ratio of Klaroid, Nup58, Nup107 and LamDm0 between the neuroblast and GMC nucleus. Bars indicate mean ± standard deviation. Number of analysed cells = 97. (**F**) Representative images of a neuroblast expressing Nup58::GFP (white on the top panel), fixed and stained for LamDm0 (green on the merge), DAPI (pink on the merge) and Miranda (yellow on the bottom panel). (**G**) Graph showing the integrated density of Mitotracker signal in the cytoplasm and in the nuclear compartment, during metaphase. Number of analysed cells = 35. Asterisks denote statistical significance, derived from unpaired t tests: ****: $p \leq 0.0001$. (**H**) Time lapse images of dividing neuroblast expressing Cherry::Jupiter (red on the merge), CD8::GFP (green on the merge) and incubated in presence of Mitotracker (white on the top panel, pink on the merges). For each experiment, the data were collected from at least 3 independent experiments. Scale bars are 5µm.

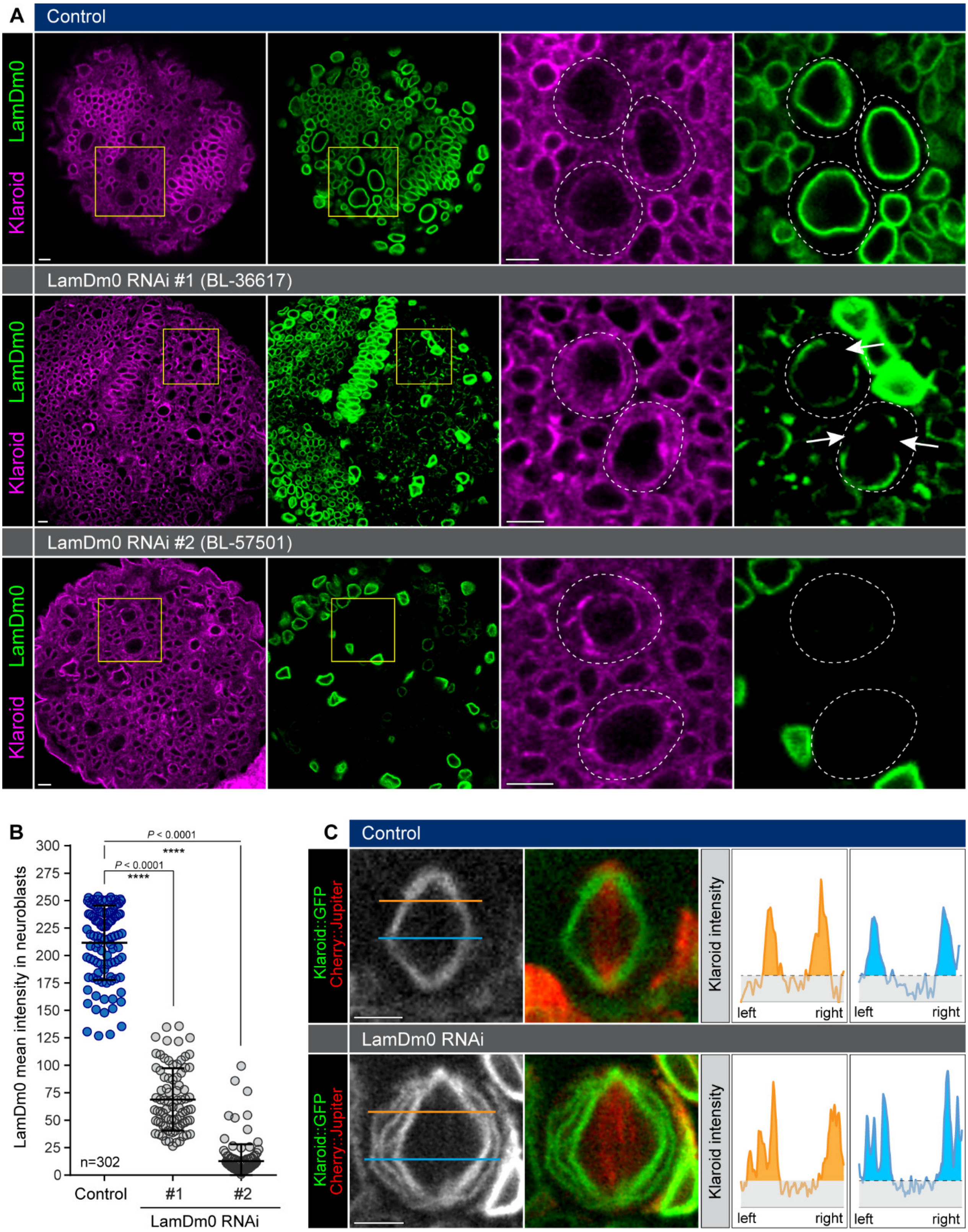
A
Control
LamDm0
Klaroid
LamDm0 RNAi #1 (BL-36617)
LamDm0 RNAi #2 (BL-57501)
B
LamDm0 mean intensity in neuroblasts
P < 0.0001
****
n=302
Control
#1
#2
LamDm0 RNAi
C
Klaroid::GFP
Cherry::Jupiter
Klaroid intensity
left
right
LamDm0 RNAi

Figure S2: A nuclear lamina is required for nuclear envelope maintenance during passage through mitosis. Related to Figure 2.

(**A**) Brain lobes expressing Klaroid (purple) in all cell types, Dicer in neuroblasts and their progeny (WorGal4 driver) (top panel) as well as an RNAi against LamDm0 in neuroblasts and their progeny (middle and bottom panels – RNAi #1 and #2, respectively), fixed and stained for LamDm0 (green). White dashed lines represent neuroblasts and white arrows show ruptures in the lamina. (**B**) Graph showing LamDm0 mean intensity along the nuclear envelope, in neuroblasts control and in neuroblasts expressing an RNAi against LamDm0 (RNAi #1 and #2). Bars indicate mean ± standard deviation. Number of analysed cells = 302. Asterisks denote statistical significance, derived from unpaired t tests: ****: $p \leq 0.0001$. (**C**) Representative images of a control neuroblast (top panel) and of a neuroblast expressing an RNAi against LamDm0 (bottom panel), expressing Klaroid::GFP (in white on the left, green on the merge) and Cherry::Jupiter (red on the merge). Graphs show plot profiles done along the orange and blue lines. For each experiment, the data were collected from at least 3 independent experiments.

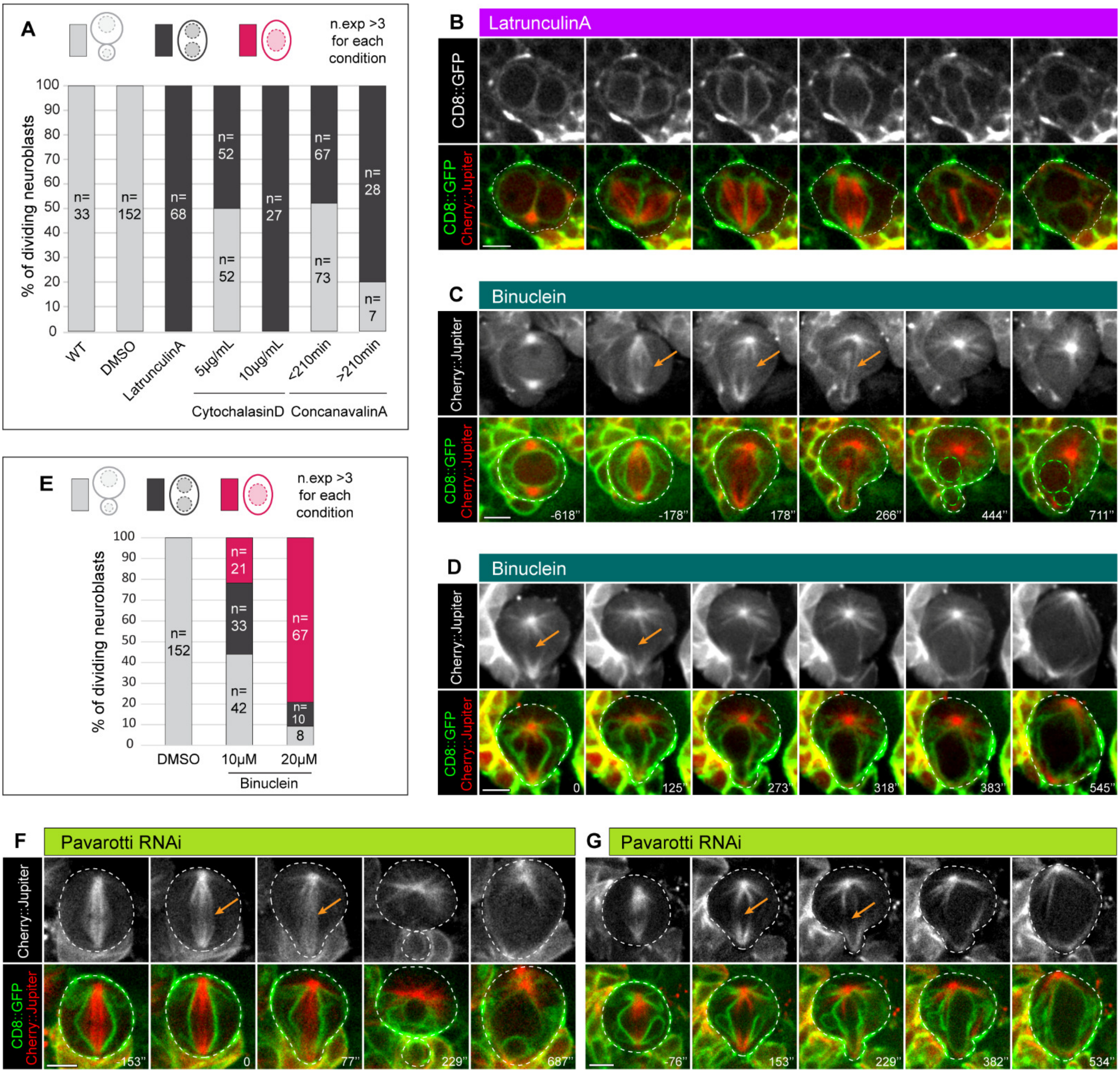

A
n.exp >3
for each
condition
% of dividing neuroblasts
100
90
80
70
60
50
40
30
20
10
0
n= 33
n= 152
n= 68
n= 52
n= 52
n= 27
n= 67
n= 73
n= 28
n= 7
WT
DMSO
LatrunculinA
5µg/mL
10µg/mL
<210min
>210min
CytochalasinD
ConcanavalinA
B
LatrunculinA
CD8::GFP
CD8::GFP Cherry::Jupiter
C
Binuclein
Cherry::Jupiter
CD8::GFP Cherry::Jupiter
-618''
-178''
178''
266''
444''
711''
D
Binuclein
Cherry::Jupiter
CD8::GFP Cherry::Jupiter
0
125''
273''
318''
383''
545''
E
n.exp >3
for each
condition
% of dividing neuroblasts
n= 152
n= 21
n= 33
n= 42
n= 67
n= 10
8
DMSO
10µM
20µM
Binuclein
F
Pavarotti RNAi
Cherry::Jupiter
CD8::GFP Cherry::Jupiter
-153''
0
77''
229''
687''
G
Pavarotti RNAi
-76''
153''
229''
382''
534''

Figure S3: Nuclear division is a sealing-dependent process. Related to Figure 3.

(**A**) Quantification of neuroblasts dividing normally (grey category), failing in cell division but still dividing their nucleus (black category) or failing in both cell and nuclear division (pink category). The number of analysed cells for each condition is indicated on the graph. (**B**) Representative timelapse images of binucleated neuroblast expressing CD8::GFP (white on the top panel, green on the merge) and Cherry::Jupiter (red on the merge), failing a second round of cell division. (**C**) Representative timelapse images of Binuclein-treated neuroblast expressing Cherry::Jupiter (white on the top panel, red on the merge) and CD8::GFP (green on the merge) failing only in cell division. Orange arrows show the central spindle. (**D**) Representative timelapse images of Binuclein-treated neuroblast expressing Cherry::Jupiter (white on the top panel, red on the merge) and CD8::GFP (green on the merge) failing in both cell and nuclear division. Orange arrows show the absence of a central spindle. (**E**) Quantification of neuroblasts dividing normally (grey category), failing in cell division but still undergoing nuclear division (black category) or failing in both cell and nuclear division (pink category) after DMSO or Binuclein treatment. The number of cells analysed for each condition is indicated on the graph. (**F, G**) Representative time lapse images of neuroblasts expressing an RNAi against Pavarotti, CD8::GFP (green on the merge) and Cherry::Jupiter (white on the top panel, red on the merge), failing only in cell division (**F**) or in both cell and nuclear division (**G**). Orange arrows show the presence (**F**) or absence (**G**) of central spindle. For each experiment, the data were collected from at least 3 independent experiments. Scale bar is 5µm.

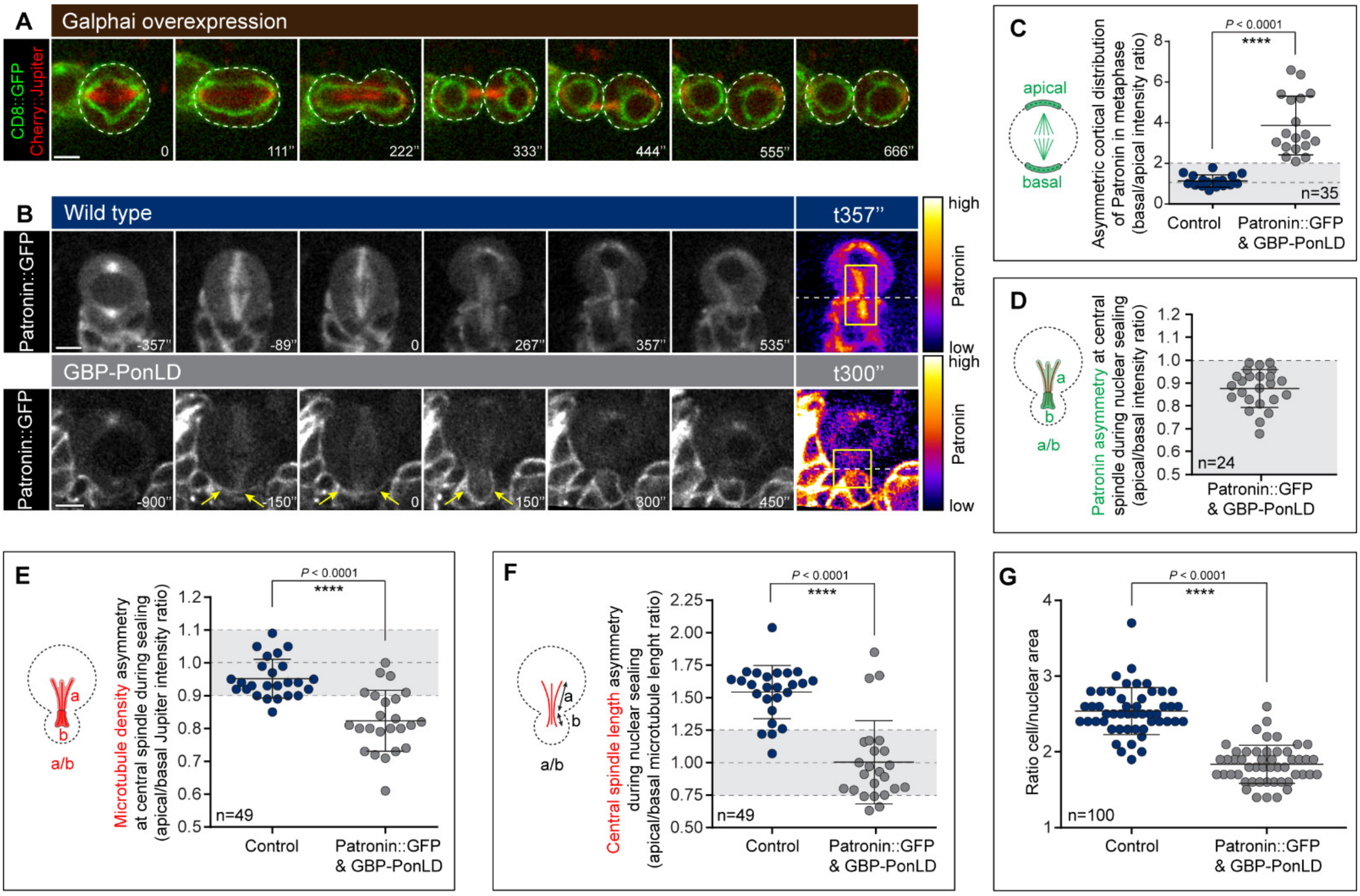

A
Galphai overexpression
CD8::GFP Cherry::Jupiter
0
111''
222''
333''
444''
555''
666''
B
Wild type
t357''
Patronin::GFP
-357''
-89''
0
267''
357''
535''
high
Patronin
low
GBP-PonLD
t300''
Patronin::GFP
-900''
-150''
0
150''
300''
450''
high
Patronin
low
C
apical
basal
Asymmetric cortical distribution of Patronin in metaphase (basal/apical intensity ratio)
P < 0.0001
****
n=35
Control
Patronin::GFP & GBP-PonLD
D
a
b
a/b
Patronin asymmetry at central spindle during nuclear sealing (apical/basal intensity ratio)
n=24
Patronin::GFP & GBP-PonLD
E
a
b
a/b
Microtubule density asymmetry at central spindle during sealing (apical/basal Jupiter intensity ratio)
P < 0.0001
****
n=49
Control
Patronin::GFP & GBP-PonLD
F
a
b
a/b
Central spindle length asymmetry during nuclear sealing (apical/basal microtubule lenght ratio)
P < 0.0001
****
n=49
Control
Patronin::GFP & GBP-PonLD
G
Ratio cell/nuclear area
P < 0.0001
****
n=100
Control
Patronin::GFP & GBP-PonLD

Figure S4: Asymmetric nuclear division depends on nuclear sealing at sites dictated by the spindle. Related to Figure 4.

(**A**) Representative time lapse images of a neuroblast overexpressing Galphai, CD8::GFP (green) and Cherry::Jupiter (red). (**B**) Representative time lapse images of a neuroblast expressing Patronin::GFP alone (top panel) or Patronin::GFP together with the localization domain of Pon fused to GFP Binding Protein (GBP-PonLD) (bottom panel). Yellow arrows show the basal recruitment of Patronin::GFP. The last pictures correspond to the sealing time (time point 357" and 300", respectively), and is shown with a gradient of colour to highlight the depletion of Patronin on the apical half of the central spindle after co-expression with GBP-PonLD. (**C**) Graph showing Patronin distribution at the apical versus basal cortex, in metaphase, in control neuroblasts and in neuroblasts expressing Patronin::GFP together with GBP-PonLD. Bars indicate mean ± standard deviation. Number of analysed cells = 35. Asterisks denote statistical significance, derived from unpaired t tests: ****: $p \leq 0.0001$. (**D**) Graph showing Patronin distribution at the apical versus basal half of the central spindle. Bars indicate mean ± standard deviation. Number of analysed cells = 24. (**E**) Graph showing the microtubule density ratio of the central spindle (apical versus basal half) in control neuroblasts and in neuroblasts expressing Patronin::GFP and GBP-PonLD. Number of analysed cells = 49. Bars indicate mean ± standard deviation. Asterisks denote statistical significance, derived from unpaired t tests: ****: $p \leq 0.0001$. (**F**) Graph showing the microtubule length ratio of the central spindle (apical versus basal half) in control neuroblasts and in neuroblasts expressing Patronin::GFP and GBP-PonLD. Number of analysed cells = 49. Bars indicate mean ± standard deviation. Asterisks denote statistical significance, derived from unpaired t tests: ****: $p \leq 0.0001$. (**G**) Graph showing the ratio between cell and nuclear size in control neuroblasts and in neuroblasts expressing Patronin::GFP and GBP-PonLD. Number of analysed cells = 100. Bars indicate mean ± standard deviation. Asterisks denote statistical significance, derived from unpaired t tests: ****: $p \leq 0.0001$. For each experiment, the data were collected from at least 3 independent experiments. Scale bars are 5µm.

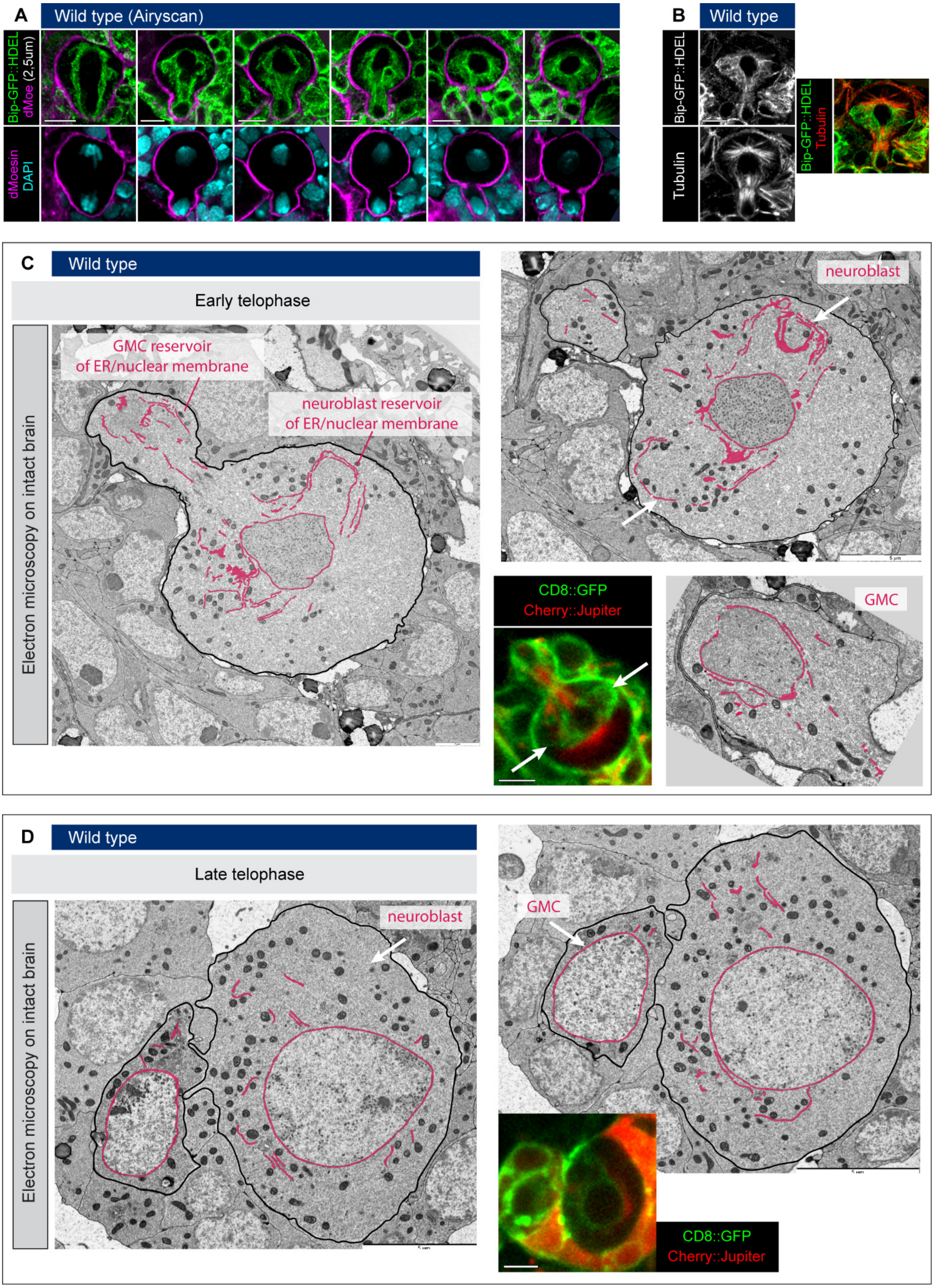
A
Wild type (Airyscan)
Bip-GFP::HDEL dMoe (2,5um)
dMoesin DAPI
B
Wild type
Bip-GFP::HDEL
Tubulin
Bip-GFP::HDEL Tubulin
C
Wild type
Early telophase
Electron microscopy on intact brain
GMC reservoir of ER/nuclear membrane
neuroblast reservoir of ER/nuclear membrane
neuroblast
CD8::GFP
Cherry::Jupiter
GMC
D
Wild type
Late telophase
Electron microscopy on intact brain
neuroblast
GMC
CD8::GFP
Cherry::Jupiter

Figure S5: Final nuclear size are achieved via differential growth of the two daughter nuclei. Related to Figure 5.

(**A**) Representative Airyscan images of neuroblasts expressing the ER marker Bip-SfGFP::HDEL (green) stained for the cortical marker dMoesin (pink) and DAPI (blue). Maximal projection of 2.5 um. (**B**) Representative Airyscan images of neuroblast expressing the ER marker Bip-SfGFP::HDEL (white on the top panel, green on the merge) stained for Tubulin (white on the bottom panel, red on the merge. (**C**) Correlative Light and Electron Microscopy showing the asymmetric partitioning of ER/nuclear membrane reservoir between the two daughter cells, during early telophase. (**D**) Correlative Light and Electron Microscopy performed in late telophase, after nuclear growth. Note the very small remaining of nuclear membranes reservoir into the neuroblast, compared with mitotic neuroblasts in late anaphase/early telophase (Figure 5F, Figure S5C).

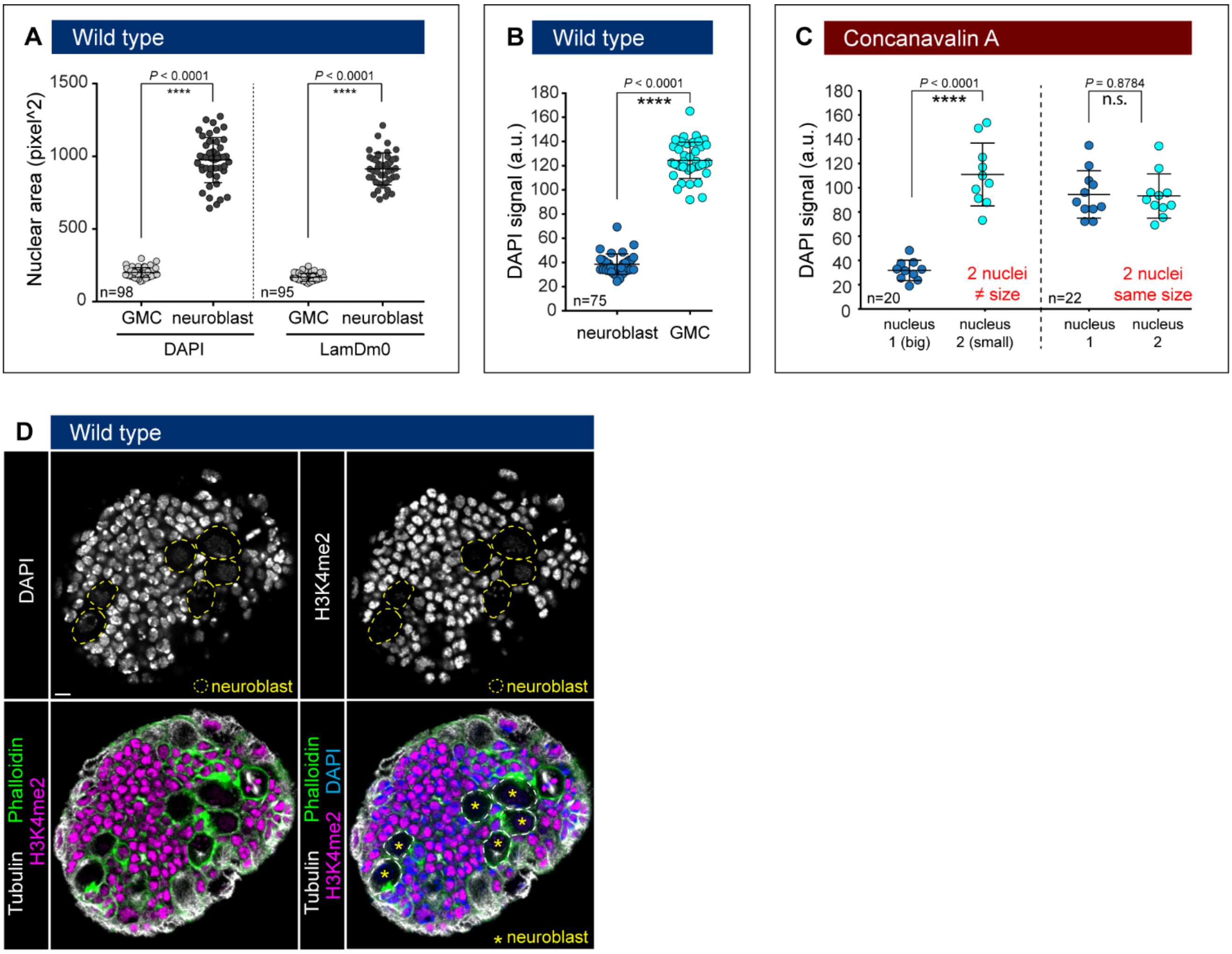

Figure S6: Asymmetric nuclear division affects chromatin organization. Related to Figure 6.

(**A**) Graph showing nuclear area of GMC and neuroblast nucleus based on DAPI (left) and LamDm0 (right) staining. Number of analysed cells = 98 and 95 for DAPI and LamDm0 staining, respectively. Bars indicate mean ± standard deviation. Asterisks denote statistical significance, derived from unpaired t tests: ****: p ≤ 0.0001. (**B**) Graph showing the DAPI mean intensity signal in the neuroblast and GMC nucleus. Number of analysed cells = 75. Bars indicate mean ± standard deviation. Asterisks denote statistical significance, derived from unpaired t tests: ****: p ≤ 0.0001. (**C**) Graph showing the DAPI mean intensity signal in sibling nuclei of binucleated neuroblasts. Number of analysed cells = 42. Bars indicate mean ± standard deviation. Asterisks denote statistical significance, derived from unpaired t tests: n.s.: not significant, ****: p ≤ 0.0001. (**D**) Wild type brain lobe fixed and stained for DAPI (white on the top left and blue on the merge), H3K4me2 (white on the top right and pink on the merges), Tubulin (white on the merges) and Phalloidin (green on the merges). Yellow dashed lines and yellow stars represent neuroblasts. For each experiment, the data were collected from at least 3 independent experiments.
